# Application of Artificial Intelligence in Community-Based Primary Health Care: Systematic Scoping Review and Critical Appraisal

**DOI:** 10.2196/29839

**Published:** 2021-09-03

**Authors:** Samira Abbasgholizadeh Rahimi, France Légaré, Gauri Sharma, Patrick Archambault, Herve Tchala Vignon Zomahoun, Sam Chandavong, Nathalie Rheault, Sabrina T Wong, Lyse Langlois, Yves Couturier, Jose L Salmeron, Marie-Pierre Gagnon, Jean Légaré

**Affiliations:** 1 Department of Family Medicine, Faculty of Medicine and Health Sciences McGill University Montreal, QC Canada; 2 Mila-Quebec AI Institute Montreal, QC Canada; 3 Department of Family Medicine and Emergency Medicine Université Laval Quebec City, QC Canada; 4 VITAM - Centre de recherche en santé durable Université Laval Quebec City, QC Canada; 5 Faculty of Engineering Dayalbagh Educational Institute Agra India; 6 Quebec SPOR-Support Unit Quebec City, QC Canada; 7 Faculty of Science and Engineering Université Laval Quebec City, QC Canada; 8 School of Nursing University of British Columbia Vancouver, BC Canada; 9 Center for Health Services and Policy Research University of British Columbia Vancouver, BC Canada; 10 Department of Industrial Relations Université Laval Quebec City, QC Canada; 11 OBVIA - Quebec International Observatory on the social impacts of AI and digital technology Quebec City, QC Canada; 12 School of Social Work University of Sherbrooke Sherbrooke, QC Canada; 13 Department of Data Science University Pablo de Olavide Seville Spain; 14 Faculty of Nursing Université Laval Quebec City, QC Canada; 15 Arthritis Alliance of Canada Montreal, QC Canada

**Keywords:** artificial intelligence, machine learning, community-based primary health care, systematic scoping review

## Abstract

**Background:**

Research on the integration of artificial intelligence (AI) into community-based primary health care (CBPHC) has highlighted several advantages and disadvantages in practice regarding, for example, facilitating diagnosis and disease management, as well as doubts concerning the unintended harmful effects of this integration. However, there is a lack of evidence about a comprehensive knowledge synthesis that could shed light on AI systems tested or implemented in CBPHC.

**Objective:**

We intended to identify and evaluate published studies that have tested or implemented AI in CBPHC settings.

**Methods:**

We conducted a systematic scoping review informed by an earlier study and the Joanna Briggs Institute (JBI) scoping review framework and reported the findings according to PRISMA-ScR (Preferred Reporting Items for Systematic Reviews and Meta-Analysis-Scoping Reviews) reporting guidelines. An information specialist performed a comprehensive search from the date of inception until February 2020, in seven bibliographic databases: Cochrane Library, MEDLINE, EMBASE, Web of Science, Cumulative Index to Nursing and Allied Health Literature (CINAHL), ScienceDirect, and IEEE Xplore. The selected studies considered all populations who provide and receive care in CBPHC settings, AI interventions that had been implemented, tested, or both, and assessed outcomes related to patients, health care providers, or CBPHC systems. Risk of bias was assessed using the Prediction Model Risk of Bias Assessment Tool (PROBAST). Two authors independently screened the titles and abstracts of the identified records, read the selected full texts, and extracted data from the included studies using a validated extraction form. Disagreements were resolved by consensus, and if this was not possible, the opinion of a third reviewer was sought. A third reviewer also validated all the extracted data.

**Results:**

We retrieved 22,113 documents. After the removal of duplicates, 16,870 documents were screened, and 90 peer-reviewed publications met our inclusion criteria. Machine learning (ML) (41/90, 45%), natural language processing (NLP) (24/90, 27%), and expert systems (17/90, 19%) were the most commonly studied AI interventions. These were primarily implemented for diagnosis, detection, or surveillance purposes. Neural networks (ie, convolutional neural networks and abductive networks) demonstrated the highest accuracy, considering the given database for the given clinical task. The risk of bias in diagnosis or prognosis studies was the lowest in the participant category (4/49, 4%) and the highest in the outcome category (22/49, 45%).

**Conclusions:**

We observed variabilities in reporting the participants, types of AI methods, analyses, and outcomes, and highlighted the large gap in the effective development and implementation of AI in CBPHC. Further studies are needed to efficiently guide the development and implementation of AI interventions in CBPHC settings.

## Introduction

The use of artificial intelligence (AI) in primary health care has been widely recommended [1]. AI systems have been increasingly used in health care, in general [2], given the hope that such systems may help develop and augment the capacity of humans in such areas as diagnostics, therapeutics, and management of patient-care and health care systems [2]. AI systems have the capability to transform primary health care by, for example, improving risk prediction, supporting clinical decision making, increasing the accuracy and timeliness of diagnosis, facilitating chart review and documentation, augmenting patient–physician relationships, and optimizing operations and resource allocation [3].

Community-based primary health care (CBPHC) is a society-wide approach to primary health care that involves a broad range of prevention measures and care services within communities, including health promotion, disease prevention and management, home care, and end-of-life care [4]. CBPHC incorporates health service delivery from personal to community levels and is the first and most frequent point of contact for the patients with health care systems for patients in many countries, including Canada [4]. In addition to providing comprehensive health care and its importance within healthcare systems, CBPHC has also been identified as essential in formulating evidence-informed public health policies [5]. Given the growing role of primary health care and CBPHC in our society [6], it is important to develop strategies that address the limitations of the existing health care system and enhance the overall quality of care delivered alongside all other aspects of CBPHC. This includes efforts for reducing the growing health care burden of CBPHC providers as well as the burden of chronic diseases, decreasing rates of misclassification and misdiagnosis, reducing cases of mismanaged diseases, and increasing accessibility to care [7-17].

Indeed, integration of AI into CBPHC could help in a variety of ways, including identifying patterns, optimizing operations, and gaining insights from clinical big data and community-level data that are beyond the capabilities of humans. Over time, using AI in CBPHC could lessen the excessive workload for health care providers by integrating large quantities of data and knowledge into clinical practice and analyzing these data in ways humans cannot, thus yielding insights that could not otherwise be obtained. This will allow health care providers to devote their time and energy to the more human aspects of health care [18]. Several studies have reported early successes of AI systems for facilitating diagnosis and disease management in different fields, including radiology [19], ophthalmology [20], cardiology [21], orthopedics [22], and pathology [23]. However, the literature also raises doubts about using and implementing AI in health care [24,25]. Aspects including privacy and consent, explainability of the algorithms, workflow disruption, and the “Frame Problem” that is defined as unintended harmful effects from issues not directly addressed for patient care [26].

Despite the potential advantages, disadvantages, and doubts, there is no comprehensive knowledge synthesis that clearly identifies and evaluates AI systems that have been tested or implemented in CBPHC. Thus, we performed a systematic scoping review aiming to (1) summarize existing studies that have tested or implemented AI methods in CBPHC; (2) report evidence regarding the effects of different AI systems’ outcomes on patients, health care providers, or health care systems, and (3) critically evaluate current studies and provide future directions for AI-CBPHC researchers.

## Methods

### Study Design

Based on the scoping review methodological framework proposed by Levac et al [27], and the Joanna Briggs Institute (JBI) methodological guidance for scoping reviews [28], we developed a protocol with the following steps: (1) clarifying the purpose of the review and linking it to a research question, (2) identifying relevant studies and balancing feasibility with breadth and comprehensiveness, (3) working in a team to iteratively select studies and extract their data, (4) charting the extracted data, incorporating a numerical summary, (5) collating, summarizing, and reporting the results, and (6) consulting the results regularly with stakeholders throughout regarding emerging and final results. This protocol is registered and available on the JBI website and the Open Science Framework (OSF) websites. We completed this review as per the published protocol.

**Figure 1:**
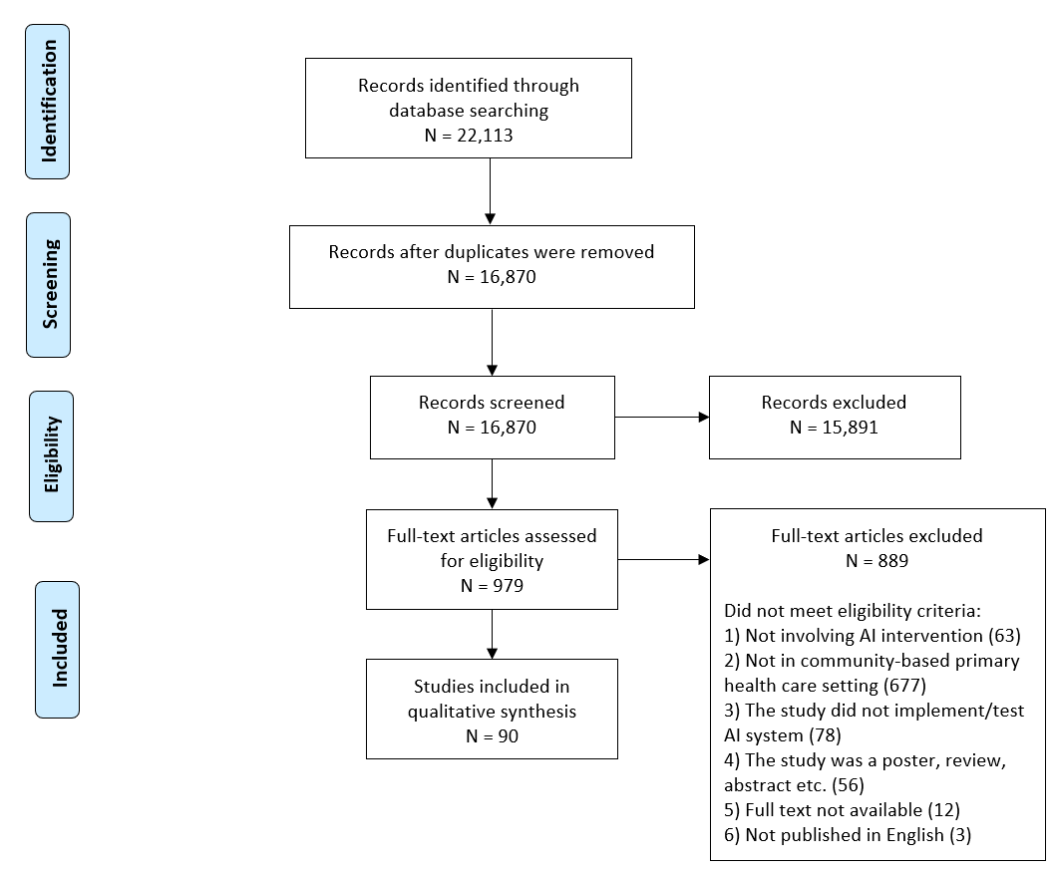
PRISMA (Preferred Reporting Items for Systematic Reviews and Meta-Analyses) flowchart of the selection procedure. AI: artificial intelligence.

We formed a multidisciplinary committee of experts in public health, primary health care, AI and data science, knowledge translation, and implementation science, as well as a patient partner and an industry partner (with expertise in the AI-health domain) with whom we consulted during all the steps of the scoping review. This helped us to interpret the results. The screening process is shown in Figure 1. Our review is reported according to the PRISMA-ScR (Preferred Reporting Items for Systematic Reviews and Meta-Analysis-Scoping Reviews) reporting guideline for reporting the study [29] (see Multimedia Appendix 1). Studies that did not report their study design are categorized by methodology according to the classification outlined by the National Institute for Health and Care Excellence [30].

We used the Prediction Model Risk of Bias Assessment Tool (PROBAST) tool for assessing the risk of bias, which includes 20 signaling questions to facilitate structured judgment of risk of bias organized in four domains of potential biases related to the following: (1) participants (covers potential sources of bias related to participant selection methods and data sources); (2) predictor variables (covers potential sources of bias related to the definition and measurement of predictors evaluated for inclusion in the model); (3) outcomes (covers potential sources of bias related to the definition and measurement of the outcomes predicted by the model); and (4) analyses (covers potential sources of bias in the statistical analysis methods) [31]. Risk of bias was judged as low, high, or unclear. If one or more domains were judged as having high risk of bias, the overall judgment was “high risk” [31].

### Eligibility Criteria

We defined our bibliographic database search strategy for peer- reviewed publications in English or French using the Population, Intervention, Comparison, Outcomes, Setting and Study (PICOS) design components [32].

### Population

Studies about any population that provides health care services, including nurses, social workers, pharmacists, dietitians, public health practitioners, physicians, and community-based workers (an unregulated type of provider) were included, as were those about any populations who receive CBPHC services. We adhered to the definition of CBPHC provided by the Canadian Institutes of Health Research (CIHR) (ie, the broad range of primary prevention measures including public health, and primary care services within the community, including health promotion and disease prevention; the diagnosis, treatment, and management of chronic and episodic illness; rehabilitation support; and end-of-life care) [4]. Studies that took place in any CBPHC points of care, including community health centers, primary care networks, clinics, and outpatient departments of hospitals, were also included. Studies conducted in emergency departments were excluded.

### Intervention

Only studies that “tested” or “implemented” or “tested and implemented” AI methods, such as computer heuristics, expert systems, fuzzy logic, knowledge representation, automated reasoning, data mining, and machine learning (eg, support vector machines, neural networks, and Bayesian networks) were included. Studies related to robot-assisted care were excluded.

### Comparison

No inclusion or exclusion criteria were considered.

### Outcomes

The primary outcomes of interest were those related to individuals receiving care (eg, cognitive outcomes, health outcomes, behavioral outcomes), providers of care (eg, cognitive outcomes, health outcomes, behavioral outcomes), and health care systems (eg, process outcomes). Moreover, we analyzed the outcomes of the AI systems for their accuracy and impact on the outcomes of care.

### Analysis Methods

All study designs using qualitative, quantitative, or mixed methods were eligible for inclusion. In particular, we included experimental and quasi-experimental studies (randomized controlled trials, quasi-randomized controlled trials, nonrandomized clinical trials, interrupted time series, and controlled before-and-after studies), and observational (cohort, case control, cross- sectional, and case series), qualitative (ethnography, narrative, phenomenological, grounded theory, and case studies), and mixed methods studies (sequential, convergent).

### Information Sources and Search Criteria

An information specialist with an epidemiologist, an AI-healthcare researcher, and a family doctor developed a comprehensive search strategy and Medical Subject Headings (MeSH) mediated by the National Library of Medicine. The systematic search was conducted from inception until February 2020 in seven bibliographic databases: Cochrane Library, MEDLINE, EMBASE, Web of Science, Cumulative Index to Nursing and Allied Health Literature (CINAHL), ScienceDirect, and IEEE Xplore. Retrieved records were managed with EndNote X9.2 (Clarivate) and imported into the DistillerSR review software (Evidence Partners, Ottawa, ON) to facilitate the selection process (see Multimedia Appendix 2 for the search strategies used on each database).

### Study Selection Process

#### Title and Abstract Screening (Level 1)

Using DistillerSR, two independent reviewers conducted a pilot screening session using a questionnaire based on our eligibility criteria to test the screening tool and to reach a common understanding. Then, the two reviewers independently screened the titles and abstracts of the remaining records. A third reviewer resolved disagreements between the two reviewers.

#### Full-Text Screening (Level 2)

Using DistillerSR and the abovementioned questionnaire, the same two reviewers independently assessed the full texts selected at level 1 for their eligibility to be included in the review. A third reviewer resolved conflicting decisions. For those references for which we did not have full-text access, we attempted to obtain access through the interlibrary loan mechanism at the McGill University Library. Studies that met the eligibly criteria were included for full data extraction.

### Data Collection

We used a data extraction form, approved by our consultative committee, that we designed based on the Cochrane Effective Practice and Organisation of Care Review Group (EPOC) data collection checklist [33]. Specifically, we extracted study characteristics (eg, design and country of the corresponding author); population characteristics (eg, number of participants and type of disease or treatment); intervention characteristics (eg, AI methods used); and outcome characteristics, including outcomes related to the patients (eg, cognitive outcomes, health outcomes, behavioral outcomes), providers of care (eg, cognitive outcomes, health outcomes, behavioral outcomes), and health care systems (eg, process outcomes).

### Assessment of Risk of Bias in the Included Studies

Two reviewers independently appraised the included studies using the criteria outlined in PROBAST to evaluate the risk of bias in each included study that was eligible for evaluation using PROBAST [31]. A third reviewer verified their appraisals.

### Synthesis

We performed a descriptive synthesis [34] to describe the studies in terms of their population (patient, primary care providers), interventions (AI systems, evaluated parameters), and outcomes. The results were arranged according to the PICOS format. The tools and techniques for developing a preliminary synthesis included textual descriptions of the studies, grouping and clustering, and tabulation.

### Consultation

Throughout the steps of the review, we regularly updated all members of the research team and requested their feedback. We also presented our preliminary results during a workshop at Université Laval, Québec, Canada, with a multidisciplinary group of experts (in public health, primary care, AI and data science, knowledge translation, implementation science, as well as a patient partner, and an industry partner) and collected their comments and feedback.

### Patient Involvement

Using a patient-centered approach, our team co-developed the protocol, conducted the review, and reported the results of this study. We integrated patients’ priorities within our research questions, search strategy terms, and outcomes of interest. Our patient partner was involved in each step of the research process, including the definition of the objectives, main analysis, descriptive synthesis, interpretation of preliminary and final results, and dissemination of the results obtained in this study.

## Results

We identified 16,870 unique records. After screening their titles and abstracts, 979 studies remained for full-text review. Ultimately, 90 studies met our inclusion criteria (Figure 1).

### Study Characteristics

#### Countries and Publication Dates

**Figure 2:**
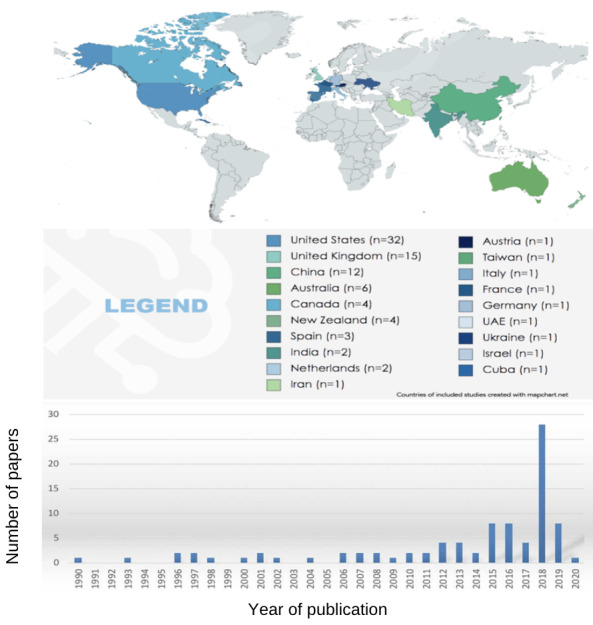
Distribution and timeline showing the publication of studies based on artificial intelligence.

The number of studies published annually has increased gradually since 1990, especially since 2015. Figure 2 shows the timeline of the AI-based studies. Moreover, the four countries publishing a high number of studies are the United States (32/90, 36%), the United Kingdom (15/90, 17%), China (12/90, 13%), and Australia (6/90, 7%). The remaining are New Zealand (4/90, 5%), Canada (4/90, 5%), Spain (3/90, 3%), India (2/90, 2%), and the Netherlands (2/90, 2%), followed by Iran, Austria, Taiwan, Italy, France, Germany, the United Arab Emirates, Ukraine, Israel, and Cuba publishing 1 study each (1%). North America accounts for the highest number of studies (37/90, 41%) followed by Europe (25/90, 28%), Asia (18/90, 20%), and Oceania (10/90, 11%).

### Aims of the Included Studies

The included studies sought to describe and test or implement either a novel AI model in CBPHC (16/90, 18%) or an off-the-shelf AI model, which is a modified or improved version of existing AI models in CBPHC (74/90, 82%).

### Conceptual Frameworks

Among the 90 studies, 2 (2%) reported using a sociocognitive theoretical framework [35,36]. One of these used the I-change model [35], a model that evolved from several cognitive models, explores the process of behavioral change and the determinants that relate to the change, and focuses on individuals’ intentions for adopting innovations [35,37]. In the first study [35] using the I-change model, the authors investigated the cognitive determinants associated with Dutch general practitioners’ intention to adopt a smoking cessation expert AI system in their respective practices and found that workload and time constraints are important barriers.

The second study used a continuing medical education framework [38] and compared traditional expert-led training (control group) with an online multimedia-based training activity supplemented with an AI-driven simulation feedback system (treatment group) [36]. Diagnosis accuracy significantly improved in the treatment group when compared to the control group, providing evidence supporting the efficacy of AI medical training methods.

### Time Frame of the Collected Data Sets

Among the included studies, 25% (23/90) used data collected over a period of 1 year or less, 20% (17/90) used data collected over a period between 1 and 5 years, 12% (11/20) used data collected over a period between 5 and 10 years, and 9% (8/90) used data collected during more than a 10-year period. One study (1%) used three data sets, collected data from three different sites with over three different time periods (<1 year, 1-5 years, >10 years) [39]. The remaining studies (30/90, 33%) did not specify the time frames of their data set collections.

### Population Characteristics

#### Patients

##### Sample Size

Overall, 88% (79/90) of the included studies reported their sample size. A total of 21,325,250 patients participated in the testing, training, or validation of the AI systems.

##### Sex, Gender, and Age

Among the 79 studies reporting their sample size, 46 (58%) reported the sex distribution and none of the studies reported on gender-relevant indicators. Further, 32 (41%) reported the participants’ mean age and standard deviation. Overall, the mean age of the participants in these studies was 60.68 (±12.15) years. Age was reported as a range in 21% (17/79) of the studies reporting the sample size, and the remaining 38% (30/79) did not report the age of their participants.

##### Ethnicity

Among all the included studies, 22% (19/79) reported the participants’ ethnic origins, which included Caucasian, Asian-Middle eastern, South Asian, African, American Indian, Alaskan Native, Hispanic, Pacific Islander, Māori, and mixed (Table 1).

**Table 1:** Characteristics of the participants in the included studies (N=90).

Participant characteristics		Value
**Patients**		
	Total number	21,325,250
	Female	2,087,374
	Male	1,814,912
	Did not report the sex	17,422,964
	Age (years), mean (SD)	60.68 (12.15)
Number of studies reporting the sample size of patients (n)		79
**Health care providers**		
	Total number	2,581
	Female	467
	Male	224
	Did not report the sex	1,890
	Age (years), mean (SD)	48.50 (7.59)
Number of studies reporting the sample size of health care providers (n)		17
	
**Ethnicities reported for patients (number)**		
	Caucasian	814,467
	Asian	8550
	African	42,057
	American Indian/Alaskan native	13
	Hispanic	5066
	Mixed ethnicity	11
	Unknown	2,241,937
	
Number of studies reporting patients’ ethnicities (n)		19
Number of studies reporting health care providers ethnicities (n)		0

##### Other Sociodemographic Information

Only 27% (25/90) of the included studies reported other sociodemographic characteristics of their participants. Socioeconomic status (ie, income level) was the most commonly reported (12/90, 13%). Other characteristics reported were educational status, marital status, area of residence, employment status, smoking status, and insurance status.

###### Health Care Providers

Among the 90 included studies, 55 (61%) reported the involvement of primary health care providers. Further, 41 of these 55 studies (75%), involved general practitioners, 5 (9%) included nurses, 1 (2%) involved psychiatrists, 1 (2%) involved occupational therapists, and 1 (2%) involved an integrated care specialist. Six studies (7%) involved general practitioners together with other types of health care providers, specifically nurses (3/55, 5%), physician assistants, (1/55 2%), nurses, surgeons, and non-surgeon specialists, (1/55, 2%) and respirologists (1/55; 2%).

##### Sample Size

Among these 55 studies, 17 (31%) reported the sample size. The data pertaining to 2581 primary health care providers were collected in these studies.

Five of these studies (29%) reported the sex distribution and none reported on gender-relevant indicators. Moreover, 2 (12%) studies reported the age of the primary health care provider participants. The mean age and SD obtained in all the studies for which we collected information is 48.50 (±7.59) years (Table 1).

##### Sociodemographic Information

Out of 17 studies, only 1 (5%) reported the primary health care providers’ locations of practice. Among the 120 providers in this study, 57 providers practiced in rural areas and 63 practiced in urban areas.

### Intervention

#### AI Methods

**Figure 3:**
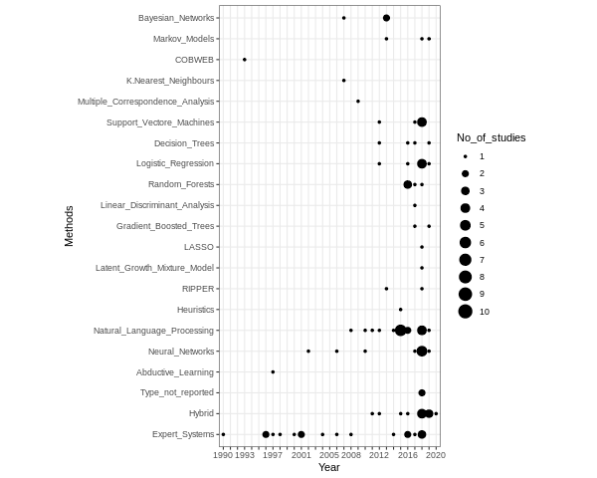
Number of studies published according to the artificial intelligence method used and years of publication.

Most of the included studies (78/90, 86%), used a single AI method (non-hybrid) and the remaining 14% (n=12) used hybrid AI models—meaning that they integrated multiple AI methods. The most commonly used methods were machine learning (ML) (41/90, 45%) and natural language processing (NLP), including applied ML for NLP (24/90, 27%), and expert systems (17/90, 19%). Figure 3 illustrates the number of studies published according to the type of AI method and year of publication (see Multimedia Appendices 3 and 4 for details regarding the AI methods).

### Performance Measures of AI Interventions

In terms of evaluating the performance of AI models, we considered the following performance metrices: True positive (TP), True negative (TN), False positive (FP), False negative (FN), sensitivity, specificity, precision, F1 score (ie, the weighted average of precision and recall, and area under the curve [AUC]). Among the 90 included studies, 31 (34%) did not report the performance of their models. Among the 59 studies that reported model performance, 13 (22%) used 2 or more performance measures and the remaining 46 (78%) used one measure (see Multimedia Appendix 4 for detailed information on studies’ AI methods used in the included studies and their performance measures).

### Generated Knowledge

Most of the included studies (81/90, 91%) were either diagnosis- or prognosis-related or focused on surveillance, and the remaining involved operational aspects (eg, resource allocation, system- level decisions) (see Multimedia Appendix 4 for detailed information).

### Health Conditions

The majority of the 90 included studies (68/90, 76%) investigated the use of AI in relation to a specific medical condition. Conditions studied were vascular diseases including hypertension, hypercholesteremia, peripheral arterial disease, and congestive heart failure (10/90, 11%) [40-49]; infectious diseases including influenza, herpes zoster, tuberculosis, urinary tract infections, and subcutaneous infections (8/90, 9%) [50-57]; type 2 diabetes (5/90, 6%) [58-62]; respiratory disorders including chronic obstructive pulmonary disease and asthma (6/90, 8%) [63-69]; orthopedic disorders including rheumatoid arthritis, gout, and lower back pain (5/90, 5%) [36,39,70-72]; neurological disorders including stroke, Parkinson disease, Alzheimer disease [73-75], and cognitive impairments (6/90, 5%) [76,77]; cancer including colorectal cancer, and head and neck cancer (4/90, 4%) [78-81]; psychological disorders including depression and schizophrenia (3/90, 3%) [82-84]; diabetic retinopathy (3/90, 3%) [85-87]; suicidal ideations (2/90, 2%) [88,89]; tropical diseases including malaria (2/90, 2%) [90,91]; renal disorders (2/90, 2%) [92,93]; autism spectrum disorder (2/90, 2%) [94,95]; venous disorders including deep vein thrombosis and venous ulcers (2/90, 2%) [96,97]; and other health conditions (8/90, 8%) [98-105].

### Data Sets (Training, Testing, and Validation)

In this section, we briefly explain the training, testing, and validation of the data sets, and then present our results. The training data set is the subset of the data that are used to fit in the initial AI model and to train it. The testing data set is the subset of the data used to evaluate the model that fits the initial training data set. The validation data set is a subset of the data used to conduct an unbiased evaluation of the model that fits the training data set, while simultaneously optimizing the model's hyperparameters, namely the parameters whose values are used to control the learning process [106]. The evaluation of these parameters is important because it provides information about the accuracy of predictions made by the AI model, and the prospective effects of hyperparameter tuning [107].

Among the 90 included studies, 9 (10%) reported on all three data sets, 33 (36%) reported on the training and testing data sets, and 36 (40%) reported on the training and validation data sets. No descriptions of these data sets were provided in 49 (54%) of the included studies.

### Legal Information and Data Privacy

Legal information concerning privacy was mentioned in 4% (4/90) of the studies in our review. Although health care records were anonymized to protect participants’ information in all four of these studies, only one explicitly reported ensuring data collection, storage, and sharing security. The remining studies did not report on data privacy and other legal information.

### Involvement of Users

#### Development

Two of the 90 included studies (2%) reported about the AI developers, all of whom were engineers [60,86]. None of the studies reported the involvement of the end users, including health care providers and patients, in the development stage.

#### Testing and Validation

Seven out of the 90 (8%) included studies reported information about those who participated in testing or validating the AI. This included general practitioners and nurses [86], engineers [60], general practitioners [51,81], occupational therapists [74], respirologists [64], and nurses [108].

### Outcomes

Extraction of the data related to the benefits for patients, primary health care providers, and the health system explained in this section was conducted according to what the authors of the included studies clearly reported as specific benefits to each of these categories.

#### Potential Benefits for Patients

Included studies reported the following potential benefits of implementing AI in CBPHC: improvements in treatment adherence, person-centered care, quality of life, timeliness of high-risk patient identification, screening speed and cost-effectiveness, enhanced predictability of morbidities and risk factors, benefits related to early diagnosis, as well as early prevention of diseases for the elderly, and facilitated referrals.

### Potential Benefits for Primary Health Care Providers

The included studies reported the following information regarding primary health care provider-related benefits of implementing AI in CBPHC: enhanced interprofessional communication and quality of primary care delivery, reduced workload of these providers, and facilitation of referrals and patient-centered care.

Other benefits included benefits with respect to use of AI as a reminder system, application of AI tools to inform commissioning health care priorities, the benefit of an AI system as a quality improvement intervention by generating warnings in electronic medical records and analyzing clinical reports, facilitating monitoring of the diseases, and using AI to reduce health risks.

### Potential Benefits for the Health Care System

Studies in our review found that AI can play a role in improving individual patient care and population-based surveillance, can be beneficial by providing predictions to inform and facilitate policy makers decisions regarding the effective management of hospitals, benefits to community-level care, cost-effectiveness, and reducing burden at the system level.

### Economic Aspects

Only one study (1%) among the included 90 papers assessed the cost-effectiveness of the AI system studied. The Predicting Out-of-Office Blood Pressure in the Clinic [PROOF-BP] system that the study authors developed for the diagnosis of hypertension in primary care was found to be cost-effective compared to conventional blood pressure diagnostic options in primary care [49].

### Challenges of Implementing AI in CBPHC

Our results suggest that challenges of using AI in CBPHC include complications related to the variability of patient data as well as barriers to use AI systems or to participate in AI research owing to the age or cognitive abilities of patients.

With respect to the health care system, our review found challenges related to how information is recorded (eg, the use of abbreviations in medical records), poor interprofessional communication between nurses and physicians, inconsistent medical tests, and a lack of event recording in cases of communication failures. The included studies also mentioned problems with respect to the restricted resources and administrative aspects such as legislations and administrative approvals, as well as challenges with respect to the lack of digital or computer literacy among the primary health care providers.

In the included studies, other challenges were reported at the level of the health care system such as the data available for use with AI as well as challenges at the level of AI itself (eg, complexity of the system and difficulty in interpretation). The following were identified as the main barriers regarding the data: (1) insufficient data to train, test, and validate AI systems, leading to negative impacts on the robustness of AI models and the accuracy of their predictions; (2) poor quality data, inaccuracies in the data, misclassifications, and lack of representative data; (3) deidentification of protected medical data; and (4) variability in the data sets and combining different data sets. Regarding AI, computational complexity and difficulties in interpreting or explaining some AI model compositions were among the barriers at the AI level.

### Risk of Bias

**Figure 4:**
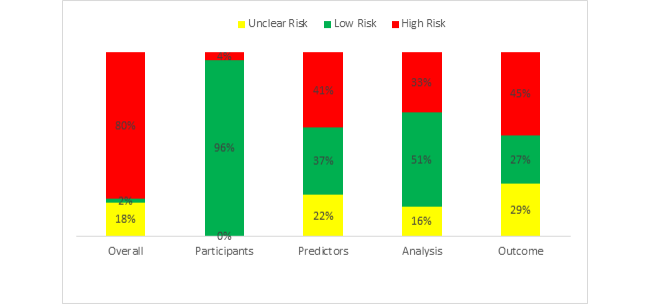
Risk of bias graph: assessing risk of bias in five categories namely overall, participants, predictors, analysis, and outcome (presented as percentages).

We identified the studies that were eligible to be evaluated using PROBAST. Among our included studies, 54% (49/90) were eligible to be evaluaeted using the PROBAST tool and most (39/49, 80%) were at high risk of bias according to our assessment with PROBAST (Figure 4). With respect to risk of bias for each of the four domains assessed, few studies presented risks regarding participants, (2/49, 4%), whereas 45% (22/49) studies exhibited risks of bias regarding outcomes. See Multimedia Appendices 5 and 6 for details on common causes of risks in each study).

## Discussion

### Principal Findings

We conducted a comprehensive systematic scoping review that included 90 studies on the use of AI systems in CBPHC and provided a critical appraisal of the current studies in this area. Our results highlighted an explosion in the number of studies since 2015. We observed variabilities in reporting the participants, type of AI methods, analysis, and outcomes, and highlighted the large gap in the effective development and implementation of AI in CBPHC. Our review led us to make the following main observations.

### AI Models, Their Performance, and Risk of Bias

ML, NLP, and expert systems were the most commonly used in CBPHC. Convolutional neural networks and abductive networks were the methods with the highest performance accuracy within the given data sets for the given task. We observed that a small number of studies reported on the development and testing or implementation of a new AI model in their study, and most of the included studies (74/90, 82%) reported on the usage and testing or implementation of an off-the-shelf AI model. Previous work has demonstrated how off-the-shelf models cannot be directly used in all clinical applications [109]. We observed a high risk of overall bias in the diagnosis- and prognosis-related studies. The highest risk of bias was in the outcome, predictor, and analysis categories of the included studies; validation of studies (external and internal) was poorly reported, and calibration was rarely assessed. A high risk of bias implies that the performance of these AI models in a new data set might not be as optimal as it was reported in these studies. Given the high risk of bias observed in the included studies, AI models used in other settings (ie, with other data) may not exhibit the same level of prediction accuracy as observed.

### Where to Use AI?

Primary health care providers are more likely to use AI systems for system-level support in administrative or health care tasks and for operational aspects, rather than for clinical making decisions [1]. However, our results show that few AI systems have been used for these purposes in CBPHC. Rather, the existing AI systems are mostly diagnosis- or prognosis-related, and used for disease detection, risk identification, or surveillance. Further studies in this regard are needed to evaluate the reason behind this tendency in addition to studies for proving the efficiency and accuracy of AI models for assisting in clinical decision making within CBPHC settings. In our review, we found that only 2 of the 90 studies used a (sociocognitive) theoretical framework. Future research needs to use knowledge, attitudes, and behavior theories to expand AI usage for clinical decision making, and more efforts are required to develop and validate frameworks guiding effective development and implementation of AI in CBPHC.

### Consideration of Age, Sex, and Gender

Our results show that AI-CBPHC research rarely considers sex, gender, age, and ethnicity. In general, the effect of age is rarely investigated in the AI field and ageism is often ignored in the analysis of discrimination. In health research, AI studies that have evaluated facial and expression recognition methods identified bias toward older adults [109]. This bias could negatively affect the accuracy of the predictions made by AI systems that are commonly used by health care providers.

Furthermore, sex and gender are sources of variations in clinical conditions, affecting different aspects including prognosis, symptomatology manifestation, and treatment effectiveness, among others [110,111]. Despite this importance, big data analytics research focusing on health through the sex and gender lens has shown that current data sets are biased given they are incomplete with respect to gender-relevant indicators with sex-disaggregated data. Indeed, less than 35% of the indicators in international databases have full disaggregation with respect to sex [112]. Our results are consistent with this observation, as we found just half of the AI-CBPHC research with patient participants and nearly one-third with health care provider participants described the sex distribution. Moreover, no AI-CBPHC research has reported on gender-relevant indicators. These are important aspects that need to be considered in the future AI-based CBPHC studies to avoid potential biases in the AI systems.

### Consideration of Ethnicity and Geographical Location

Less than one quarter of included studies have reported patient participants’ ethnicities, with no discussion on the ethnicities of participating health care providers. Moreover, for those studies that reported patient ethnicity, we observed that the collected data were related to causation populations, thus raising questions regarding the representativeness of the data set, leading to biases. Such biases could result in the AI system making predictions that discriminate against marginalized and vulnerable patient populations, ultimately leading to undesirable patient outcomes.

According to our results, most of the AI research in CBPHC has taken place in North American and Europe-centric settings. Several factors contribute to ethnoracial biases when using AI, including not accounting for ethnoracial information, thereby ignoring the different effects illnesses can have on different populations [113]. Consequently, studies can yield results with historical biases as well as biases related to over- or under-representation of population characteristics in data sets and in the knowledge, bases used to build AI systems. In turn, stereotypes and undesirable outcomes may be amplified. Ensuring ethnic diversity in study populations and accounting for this diversity in analyses is an imperative for developing AI systems that result in equitable CBPHC.

### Involvement of Users

Despite the many potential benefits of AI to humans, the development of AI systems is often based on “technology-centered” design approaches instead of "human-centered" approaches [114]. Our results indicate that no AI-CBPHC study has involved any end users in the system development stage and involving primary health care professional users during the validation or testing stages has been rare. This results in AI systems that do not meet the needs of health care providers and patients; they suffer from poor usage scenarios and eventually fail during implementation in clinical practice. A recent assessment of the current user-centered design methods showed that most of the existing user-centered design methods were primarily created for non-AI systems and do not effectively address the unique issues in AI systems [115]. Further efforts are needed to include health care providers and patients as users of the developed AI systems in the design, development, validation, and implementation stages in CBPHC. Nevertheless, effectively involving these users in the development, testing, and validation of AI systems remains a challenge; further studies are required to overcome them.

### Ethical and Legal Aspects

Ethical and legal challenges related to the use of AI in health care include, but are not limited to, informed consent to use AI, safety and transparency of personal data, algorithmic fairness, influenced by the aforementioned biases, liability, data protection, and data privacy. Our results indicate that ethical and legal aspects have rarely been addressed in AI-CBPHC research, except with respect to privacy and data security issues. There is a need to address all legal and ethical aspects and considerations within AI-CBPHC studies to facilitate implementation of AI in CBPHC settings. For instance, to increase the use of AI systems by CBPHC providers, clarifying scenarios in which informed consent is required could be useful, as would clarifying providers’ responsibilities regarding the use of AI systems. To improve patient outcomes related to AI use in CBPHC, defining the responsibilities of providers and researchers regarding the development and implementation of AI-health literacy programs for patients may be necessary, together with gaining an understanding of how and when patients need to be informed about the results that AI systems yield.

### Economic Aspects

AI systems can provide solutions to rising health care costs; however, only one (1%) AI-CBPHC study has addressed this issue by conducting a cost-effectiveness analysis of AI use. This is consistent with other study results showing that the cost-effectiveness of using AI in health care is rarely and inadequately reported [116,117]. Thus, further research analyzing cost-effectiveness is needed for identifying the economic benefits of AI in CBPHC in terms of treatment, time and resource management, and mitigation of human error; this would be valuable as it could influence decisions for or against implementing AI in CBPHC.

### AI in Clinical Practice

Our results show different barriers and facilitators for implementing AI in clinical practice. Aspects related to the data were among mostly mentioned ones. For instance, the lack of high amounts of quality data, specifically when using modern AI methods (eg, deep learning), is a challenge commonly faced when developing AI systems for use in CBPHC. The promotion of AI-driven innovation in any setting, including CBPHC, is closely linked to data governance, open data directives, and other data initiatives, as they help to establish trustworthy mechanisms and services for sharing, reusing, and pooling data [118] that are required for the development of high-quality data-driven AI systems.

In addition, some data security and privacy laws can create a bottleneck, limiting the use of AI systems in CBPHC and the sharing of health care information that is required for developing high- performance AI systems. To facilitate the implementation and adoption of high-quality AI systems in CBPHC and ensuring benefits to patients, providers and the health care system, research providing insights for addressing these implementation challenges is needed.

### Limitations of the Study

Our review has some limitations. Firstly, given that we used the Canadian Institute of Health Research’s definition of CBPHC to determine our inclusion criteria and given that the definition of CBPHC differs from one country to another, our search strategy may not have captured all relevant records. Secondly, we excluded studies conducted in emergency care settings. In many countries, emergency departments are the points of access to community-based care. The European Commission recently released a legal framework (risk-based approach) for broad AI governance among EU member states [118] and categorized emergency care and first aid services as “high risk.” Requirements of high-quality data, documentation and traceability, transparency, human oversight, and model accuracy and robustness are cited as being strictly necessary to mitigate the risks in these settings [118].

### Conclusion

In this systematic scoping review, we have demonstrated the extent and variety of AI systems being tested and implemented in CBPHC, critically evaluated these AI systems, showed that this field is growing exponentially, and exposed knowledge gaps that remain and that should be prioritized in future studies.

## Appendix

Multimedia Appendix 1 PRISMA-ScR (Preferred Reporting Items for Systematic Reviews and Meta-Analyses extension for Scoping Reviews) checklist.

Multimedia Appendix 2 Full search strategy.

Multimedia Appendix 3 Timeline of artificial intelligence implementation in community-based primary health care between 1990 and 2020.

Multimedia Appendix 4 Data extracted from the included studies.

Multimedia Appendix 5 Details on the risk of bias in each evaluated study.

Multimedia Appendix 6 Risk of bias graph based on authors’ judgments about each risk of bias item presented as percentages.

## Abbreviations

AI: Artificial Intelligence
CBPHC: Community-Based Primary Health Care
CIHR: Canadian Institutes of Health Research
CINAHL: Cumulative Index to Nursing and Allied Health Literature
EPOC: Cochrane Effective Practice and Organisation of Care Review Group
FN: False negative
FP: False positive
JBI: Joanna Briggs Institute
MeSH: Medical Subject Headings
ML: Machine Learning
PICOS: Population, Intervention, Comparison, Outcomes, Setting and Study designs
PRISMA-ScR: Preferred Reporting Items for Systematic Reviews and Meta-Analysis-Scoping Reviews
PROBAST: Prediction Model Risk of Bias Assessment Tool
NLP: Natural Language Processing
OSI: Open Science Framework
TN: True negative
TP: True positive
