# Supplementary material for: Application of Artificial Intelligence in Community-Based Primary Health Care: Systematic Scoping Review and Critical Appraisal
Source: J Med Internet Res. 2021 Sep 3;23(9):e29839. doi: 10.2196/29839 (PMC8449300; doi:10.2196/29839)
Supplement: Multimedia Appendix 2 [file jmir_v23i9e29839_app2.pdf]

**Multimedia Appendix 2.** Full search strategy.

## Table of contents :

Medline (2020-02-07) .......... 2
Cochrane Library (2020-02-07) .......... 5
Embase (2020-02-07) .......... 9
Web of Science (2020-02-07) .......... 12
Cinahl (2020-02-10) .......... 13
IEE Explore (2020-02-07) .......... 16
Science Direct (2020-02-14) .......... 22

# Medline (2020-02-07)

| PICOSS | Concepts | Research strategy keywords | Research | # Results |
|---|---|---|---|---|
| Population | Artificial Intelligence (Controlled Vocabulary) | Artificial Intelligence/ OR DATA MINING/ OR EXPERT SYSTEMS/ OR fuzzy logic/ OR exp Machine Learning/ OR NATURAL LANGUAGE PROCESSING/ OR Support Vector Machine/ OR "neural networks (computer)"/ | #1 | 72 024 |
| | Artificial Intelligence (Free text) | (artificial intelligence*" or "computational intelligence*" or "machine intelligence*" or "automated reasoning" or "bayesian network*" or "bayes network*" or "naive bayes" or "bayesian learning" or "computer heuristic*" or "computer reasoning" or "data mining" or "text mining" or "expert system*" or "fuzzy logic" or "fuzzy cognitive" or "knowledge representation*" or "knowledge acquisition*" or "machine learning" or "learning machine*" or "natural language processing*" or "neural network*" or "deep learning" or "support vector*" or "hidden markov model*" or "random forest*" or "random decision forest*" or "supervised learning" or "unsupervised learning" or "autoencoder*" or "Generative adversarial network*" or "reservoir computing" or "shallow learning" or "echo state network*" or "case-based reasoning" or "metaheuristic*" or "soft computing" or "approximate reasoning" or "evolutionary computing" or "genetic algorithm*" or "bio-inspired algorithm*").ti.<br><br>or ("artificial intelligence*" or "computational intelligence*" or "machine intelligence*" or "automated reasoning" or "bayesian network*" or "bayes network*" or "naive bayes" or "bayesian learning" or "computer heuristic*" or "computer reasoning" or "data mining" or "text mining" or "expert system*" or "fuzzy logic" or "fuzzy cognitive" or "knowledge representation*" or "knowledge acquisition*" or "machine learning" or "learning machine*" or "natural language processing*" or "neural network*" or "deep learning" or "support vector*" or "hidden markov model*" or "random forest*" or "random decision forest*" or "supervised learning" or "unsupervised learning" or "autoencoder*" or "Generative adversarial network*" or "reservoir computing" or "shallow learning" or "echo state network*" or "case-based reasoning" or "metaheuristic*" or "soft computing" or "approximate reasoning" or "evolutionary computing" or "genetic algorithm*" or "bio-inspired algorithm*").ab.<br><br>or ("artificial intelligence*" or "computational intelligence*" or "machine intelligence*" or "automated reasoning" or "bayesian network*" or "bayes network*" or "naive bayes" or "bayesian learning" or "computer heuristic*" or "computer reasoning" or "data mining" or "text mining" or "expert system*" or "fuzzy logic" or "fuzzy cognitive" or "knowledge representation*" or "knowledge acquisition*" or "machine learning" or "learning machine*" or "natural language processing*" or "neural network*" or "deep learning" or "support vector*" or "hidden markov model*" or "random forest*" or "random decision forest*" or "supervised learning" or "unsupervised learning" or "autoencoder*" or "Generative | #2 | 115 253 |

| | | | | |
|---|---|---|---|---|
| | | adversarial network*" or "reservoir computing" or "shallow learning" or "echo state network*" or "case-based reasoning" or "metaheuristic*" or "soft computing" or "approximate reasoning" or "evolutionary computing" or "genetic algorithm*" or "bio-inspired algorithm*").kw. | | |
| | Artificial Intelligence | #1 OR #2 | #3 | 144 526 |
| Settings | Community-Based Primary Health Care (Controlled vocabulary | Primary Health Care/ or exp General Practice/ or Physicians, Family/ or General Practitioners/ or Physicians, Primary Care/ or exp Group Practice/ or Ambulatory Care/ or exp Community Health Services/ or exp Ambulatory Care Facilities/ or exp Rural Health Services/ or exp nurse practitioners/ or Community Health Workers/ or Community Medicine/ or home nursing/ or primary care nursing/ or nurses, community health/ or nurses, public health/ or nurse clinicians/ or nurse midwives/ or Pharmacists/ or exp Home Care Services/ or Hospices/ or exp Community Health Nursing/ or Office Visits/ or House Calls/ or emergency medical technicians/ or exp Emergency Service, Hospital/ or pharmacy technicians/ OR emergency medical services/ or emergency services, psychiatric/ OR Family Health/ | #4 | 719 520 |
| | Community-Based Primary Health Care (Free text) | ((primary adj3 care) or ("primary healthcare" or "primary health" or "first line" or "family healthcare") or ((family or general* or group) adj2 (doctor* or physician* or pract* or medicine or nurs*)) or (rural adj3 (physician* or practice or service* or hospital*)) or generalist* or (ambulatory adj2 (care or clinic* or service*)) or (health adj3 (center* or centre*)) or consult* or (visit* adj3 (clinic* or care or outpatient* or office*)) or (community adj3 (care or worker* or service* or nurs* or pract*)) or (social adj2 (worker* or service*)) or practitioner* or "clinical practice*" or pharmacist* or dietitian* or hospice* or ((home or domicil*) adj3 (care or healthcare or nurs* or rehabilit* or service* or visiting or visit?)) or homecare* or (nurse adj1 (rehabilitator* or clinician*)) or paramedic* or ((emergency or emergencies or trauma) adj3 (department* or service* or outpatient* or ward* or room* or unit or units or center* or centre* or physician* or nurse*)) or "ED" or "family health" or midwi*).ti.<br><br>or ((primary adj3 care) or ("primary healthcare" or "primary health" or "first line" or "family healthcare") or ((family or general* or group) adj2 (doctor* or physician* or pract* or medicine or nurs*)) or (rural adj3 (physician* or practice or service* or hospital*)) or generalist* or (ambulatory adj2 (care or clinic* or service*)) or (health adj3 (center* or centre*)) or consult* or (visit* adj3 (clinic* or care or outpatient* or office*)) or (community adj3 (care or worker* or service* or nurs* or pract*)) or (social adj2 (worker* or service*)) or practitioner* or "clinical practice*" or pharmacist* or dietitian* or hospice* or ((home or domicil*) adj3 (care or healthcare or nurs* or rehabilit* or service* or visiting or visit?)) or homecare* or (nurse adj1 (rehabilitator* or clinician*)) or paramedic* or ((emergency or emergencies or trauma) adj3 (department* or service* or outpatient* or ward* or room* or unit or units or center* or centre* or physician* or nurse*)) or "ED" or "family health" or midwi*).ab. | #5 | 1 059 780 |

| | | | | |
|---|---|---|---|---|
| | | or ((primary adj3 care) or ("primary healthcare" or "primary health" or "first line" or "family healthcare") or ((family or general* or group) adj2 (doctor* or physician* or pract* or medicine or nurs*)) or (rural adj3 (physician* or practice or service* or hospital*)) or generalist* or (ambulatory adj2 (care or clinic* or service*)) or (health adj3 (center* or centre*)) or consult* or (visit* adj3 (clinic* or care or outpatient* or office*)) or (community adj3 (care or worker* or service* or nurs* or pract*)) or (social adj2 (worker* or service*)) or practitioner* or "clinical practice*" or pharmacist* or dietitian* or hospice* or ((home or domicil*) adj3 (care or healthcare or nurs* or rehabilit* or service* or visiting or visit?)) or homecare* or (nurse adj1 (rehabilitator* or clinician*)) or paramedic* or ((emergency or emergencies or trauma) adj3 (department* or service* or outpatient* or ward* or room* or unit or units or center* or centre* or physician* or nurse*)) or "ED" or "family health" or midwi*).kw. | | |
| | Primary Health Care | #4 OR #5 | #6 | 1 470 958 |
| Total result | | #3 AND #6 | #7 | 5 762 |
| | Conference | limit 7 to (clinical conference or congresses or consensus development conference or consensus development conference, nih) | #8 | 8 |
| | Total result with filter for conference | #7 NOT #8 | #9 | 5 754 |

# Cochrane Library (2020-02-07)

| PICOSS | Concepts | Research strategy keywords | Research | # Results |
|---|---|---|---|---|
| Population | Artificial Intelligence (Controlled Vocabulary) | MeSH descriptor: [Artificial Intelligence] explode all trees | #1 | 998 |
| | | MeSH descriptor: [Data Mining] explode all trees | #2 | 19 |
| | | MeSH descriptor: [Fuzzy Logic] explode all trees | #3 | 37 |
| | | MeSH descriptor: [Neural Networks (Computer)] explode all trees | #4 | 109 |
| | | #1 or #2 or #3 or #4 | #5 | 1 016 |
| | Artificial Intelligence (Free text) | ("artificial intelligence*" or "computational intelligence*" or "machine intelligence*" or "automated reasoning" or "bayesian network*" or "bayes network*" or "naive bayes" or "bayesian learning" or "computer heuristic*" or "computer reasoning" or "data mining" or "text mining" or "expert system*" or "fuzzy logic" or "fuzzy cognitive" or "knowledge representation*" or "knowledge acquisition*" or "machine learning" or "learning machine*" or "natural language processing*" or "neural network*" or "deep learning" or "support vector*" or "hidden markov model*" or "random forest*" or "random decision forest*" or "supervised learning" or "unsupervised learning" or "autoencoder*" or "Generative adversarial network*" or "reservoir computing" or "shallow learning" or "echo state network*" or "case-based reasoning" or "metaheuristic*" or "soft computing" or "approximate reasoning" or "evolutionary computing" or "genetic algorithm*" or "bio-inspired algorithm*"):ti,ab,kw | #6 | 3 042 |
| | Artificial Intelligence | #5 or #6 | #7 | 3 691 |
| | Community-Based Primary Health Care (Controlled vocabulary | MeSH descriptor: [Primary Health Care] explode all trees | #8 | 6 949 |

| PICOSS | Concepts | Research strategy keywords | Research | # Results |
|---|---|---|---|---|
| | | MeSH descriptor: [Physicians, Primary Care] explode all trees | #9 | 155 |
| | | MeSH descriptor: [Primary Care Nursing] explode all trees | #10 | 30 |
| | | MeSH descriptor: [Primary Nursing] explode all trees | #11 | 24 |
| | | MeSH descriptor: [Hospitals, General] explode all trees | #12 | 143 |
| | | MeSH descriptor: [General Practitioners] explode all trees | #13 | 258 |
| | | MeSH descriptor: [General Practice] explode all trees | #14 | 2 429 |
| | | MeSH descriptor: [Family Health] explode all trees | #15 | 432 |
| | | MeSH descriptor: [Physicians, Family] explode all trees | #16 | 449 |
| | | MeSH descriptor: [Group Practice] explode all trees | #17 | 328 |
| | | MeSH descriptor: [Ambulatory Care] explode all trees | #18 | 3 598 |
| | | MeSH descriptor: [Ambulatory Care Facilities] explode all trees | #19 | 1 819 |
| | | MeSH descriptor: [Community Health Workers] explode all trees | #20 | 453 |

| PICOSS | Concepts | Research strategy keywords | Research | # Results |
|---|---|---|---|---|
| | | MeSH descriptor: [Community Health Centers] explode all trees | #21 | 559 |
| | | MeSH descriptor: [Community Health Nursing] explode all trees | #22 | 344 |
| | | MeSH descriptor: [Community Health Services] explode all trees | #23 | 13 246 |
| | | MeSH descriptor: [Rural Health Services] explode all trees | #24 | 340 |
| | | MeSH descriptor: [Hospitals, Rural] explode all trees | #25 | 36 |
| | | MeSH descriptor: [Home Nursing] explode all trees | #26 | 290 |
| | | MeSH descriptor: [Nurse Clinicians] explode all trees | #27 | 155 |
| | | MeSH descriptor: [Nurse Midwives] explode all trees | #28 | 99 |
| | | MeSH descriptor: [Nurse Practitioners] explode all trees | #29 | 297 |
| | | MeSH descriptor: [Community Medicine] explode all trees | #30 | 35 |
| | | MeSH descriptor: [Pharmacists] explode all trees | #31 | 562 |
| | | MeSH descriptor: [Pharmacy Technicians] explode all trees | #32 | 17 |
| | | MeSH descriptor: [Office Visits] explode all trees | #33 | 446 |

| PICOSS | Concepts | Research strategy keywords | Research | # Results |
|---|---|---|---|---|
| | | MeSH descriptor: [House Calls] explode all trees | #34 | 499 |
| | | MeSH descriptor: [Emergency Medical Services] explode all trees | #35 | 3 734 |
| | | MeSH descriptor: [Emergency Medical Technicians] explode all trees | #36 | 165 |
| | | MeSH descriptor: [Emergency Service, Hospital] explode all trees | #37 | 2 276 |
| | | MeSH descriptor: [Emergency Services, Psychiatric] explode all trees | #38 | 49 |
| Settings | | #8 or #9 or #10 or #11 or #12 or #13 or #14 or #15 or #16 or #17 or #18 or #19 or #20 or #21 or #22 or #23 or #24 or #25 or #26 or #27 or #28 or #29 or #30 or #31 or #32 or #33 or #34 or #35 or #36 or #37 or #38 | #39 | 30 466 |
| | Community-Based Primary Health Care (Free text) | ((primary NEAR/3 care) or ("primary healthcare" or "primary health" or "first line" or "family healthcare") or ((family or general* or group) NEAR/2 (doctor* or physician* or pract* or medicine or nurs*)) or (rural NEAR/3 (physician* or practice or service* or hospital*)) or generalist* or (ambulatory NEAR/2 (care or clinic* or service*)) or (health NEAR/3 (center* or centre*)) or consult* or (visit* NEAR/3 (clinic* or care or outpatient* or office*)) or (community NEAR/3 (care or worker* or service* or nurs* or pract*)) or (social NEAR/2 (worker* or service*)) or practitioner* or "clinical practice*" or pharmacist* or dietitian* or hospice* or ((home or domicil*) NEAR/3 (care or healthcare or nurs* or rehabilit* or service* or visiting or visit?)) OR homecare* or (nurse NEAR/1 (rehabilitator* or clinician*)) or paramedic* or ((emergency or emergencies or trauma) NEAR/3 (department* or service* or outpatient* or ward* or room* or unit or units or center* or centre* or physician* or nurse*)) or "ED" or "family health" or midwi*):ti,ab,kw | #40 | 150 586 |
| | Community-Based Primary Health Care | #39 or #40 | #41 | 158 838 |
| Total result | | #7 and #41 | #42 | 466 |
| | 2000-2018 | Retrieved manually in Endnote | #43 | 398 |

# Embase (2020-02-07)

| PICOSS | Concepts | Research strategy keywords | Research | # Results |
|---|---|---|---|---|
| Population | Artificial Intelligence (Controlled Vocabulary) | 'artificial intelligence'/exp OR 'artificial intelligence' OR 'machine learning'/exp OR 'machine learning' OR 'expert system'/exp OR 'expert system' OR 'fuzzy logic'/exp OR 'fuzzy logic' OR 'natural language processing'/exp OR 'natural language processing' OR 'support vector machine'/exp OR 'support vector machine' OR 'computational intelligence'/exp OR 'computational intelligence' OR 'bayesian network'/exp OR 'bayesian network' OR 'bayesian learning'/exp OR 'bayesian learning' OR 'text mining'/exp OR 'text mining' OR 'fuzzy cognitive map'/exp OR 'fuzzy cognitive map' OR 'deep learning'/exp OR 'deep learning' OR 'hidden markov model'/exp OR 'hidden markov model' OR 'autoencoder'/exp OR 'autoencoder' OR 'echo state network'/exp OR 'echo state network' OR 'case based reasoning'/exp OR 'case based reasoning' OR 'genetic algorithm'/exp OR 'genetic algorithm' | #1 | 231 712 |
| | Artificial Intelligence (Free text) | ("artificial intelligence*" or "computational intelligence*" or "machine intelligence*" or "automated reasoning" or "bayesian network*" or "bayes network*" or "naive bayes" or "bayesian learning" or "computer heuristic*" or "computer reasoning" or "data mining" or "text mining" or "expert system*" or "fuzzy logic" or "fuzzy cognitive" or "knowledge representation*" or "knowledge acquisition*" or "machine learning" or "learning machine*" or "natural language processing*" or "neural network*" or "deep learning" or "support vector*" or "hidden markov model*" or "random forest*" or "random decision forest*" or "supervised learning" or "unsupervised learning" or "autoencoder*" or "Generative adversarial network*" or "reservoir computing" or "shallow learning" or "echo state network*" or "case-based reasoning" or "metaheuristic*" or "soft computing" or "approximate reasoning" or "evolutionary computing" or "genetic algorithm*" or "bio-inspired algorithm*"):ti,ab,kw | #2 | 151 354 |
| | Artificial Intelligence | #1 OR #2 | #3 | 265 536 |
| Settings | Community-Based Primary Health Care (Controlled vocabulary | 'primary health care'/exp OR 'primary health care' OR 'general practice'/exp OR 'general practice' OR 'general practitioner'/exp OR 'general practitioner' OR 'ambulatory care'/exp OR 'ambulatory care' OR 'community mental health center'/exp OR 'community mental health center' OR 'rural health care'/exp OR 'rural health care' OR 'rural health centers'/exp OR 'rural health centers' OR 'nurse practitioner'/exp OR 'nurse practitioner' OR 'group practice'/exp OR 'group practice' OR 'health center'/exp OR 'health center' OR 'community medicine'/exp OR 'community medicine' OR 'clinical nurse specialist'/exp OR 'clinical nurse | #4 | 1 528 357 |

| | | | | |
|---|---|---|---|---|
| | | specialist' OR 'nurse midwife'/exp OR 'nurse midwife' OR 'pharmacist'/exp OR 'pharmacist' OR 'outpatient department'/exp OR 'outpatient department' OR 'hospice'/exp OR 'hospice' OR 'community health nursing'/exp OR 'community health nursing' OR 'home visit'/exp OR 'home visit' OR 'paramedical personnel'/exp OR 'paramedical personnel' OR 'emergency health service'/exp OR 'emergency health service' OR 'emergency ward'/exp OR 'emergency ward' OR 'pharmacy technician'/exp OR 'pharmacy technician' OR 'family health'/exp OR 'family health' OR 'community care'/exp OR 'community care' OR 'social worker'/exp OR 'social worker' OR 'dietitian'/exp OR 'dietitian' OR 'home care'/exp OR 'home care' | | |
| | Community-Based Primary Health Care (Free text) | (primary NEAR/3 care):ti,ab,kw or ("primary healthcare" or "primary health" or "first line" or "family healthcare"):ti,ab,kw or ((family or general* or group) NEAR/2 (doctor* or physician* or pract* or medicine or nurs*)):ti,ab,kw or (rural NEAR/3 (physician* or practice or service* or hospital*)):ti,ab,kw or generalist*:ti,ab,kw or (ambulatory NEAR/2 (care or clinic* or service*)):ti,ab,kw or (health NEAR/3 (center* or centre*)):ti,ab,kw or consult*:ti,ab,kw or (visit* NEAR/3 (clinic* or care or outpatient* or office*)):ti,ab,kw or (community NEAR/3 (care or worker* or service* or nurs* or pract*)):ti,ab,kw or (social NEAR/2 (worker* or service*)):ti,ab,kw or practitioner*:ti,ab,kw or "clinical practice*":ti,ab,kw or pharmacist*:ti,ab,kw or dietitian*:ti,ab,kw or hospice*:ti,ab,kw or ((home or domicil*) NEAR/3 (care or healthcare or nurs* or rehabilit* or service* or visiting or visit or visits)):ti,ab,kw or homecare*:ti,ab,kw or (nurse NEAR/1 (rehabilitator* or clinician*)):ti,ab,kw or paramedic*:ti,ab,kw or ((emergency or emergencies or trauma) NEAR/3 (department* or service* or outpatient* or ward* or room* or unit or units or center* or centre* or physician* or nurse*)):ti,ab,kw or "ED":ti,ab,kw or "family health":ti,ab,kw or midwi*:ti,ab,kw | #5 | 1 595 478 |
| | Primary Health Care | #4 OR #5 | #6 | 2 435 012 |
| | Total result | #3 AND #6 | #7 | 12 994 |
| | Conference | limit 7 to('conference abstract'/it OR 'conference paper'/it OR 'conference review'/it) | #8 | 3 146 |
| | | #7 NOT #8 | #9 | 9 848 |

| | | | | |
|---|---|---|---|---|
| | Sources | #9 AND [embase]/lim NOT ([embase]/lim AND [medline]/lim) | #10 | 1 607 |

# Web of Science (2020-02-07)

| PICOSS | Concepts | Research strategy keywords | Research | # Results |
|---|---|---|---|---|
| Population | Artificial Intelligence (Free text) | TS=("artificial intelligence*" or "computational intelligence*" or "machine intelligence*" or "automated reasoning" or "bayesian network*" or "bayes network*" or "naive bayes" or "bayesian learning" or "computer heuristic*" or "computer reasoning" or "data mining" or "text mining" or "expert system*" or "fuzzy logic" or "fuzzy cognitive" or "knowledge representation*" or "knowledge acquisition*" or "machine learning" or "learning machine*" or "natural language processing*" or "neural network*" or "deep learning" or "support vector*" or "hidden markov model*" or "random forest*" or "random decision forest*" or "supervised learning" or "unsupervised learning" or "autoencoder*" or "Generative adversarial network*" or "reservoir computing" or "shallow learning" or "echo state network*" or "case-based reasoning" or "metaheuristic*" or "soft computing" or "approximate reasoning" or "evolutionary computing" or "genetic algorithm*" or "bio-inspired algorithm*")<br>Indexes=SCI-EXPANDED, SSCI, A&HCI, ESCI Timespan=All years | #1 | 505 123 |
| Settings | Community-Based Primary Health Care (Free text) | TS=((primary NEAR/2 care) or ("primary healthcare" or "primary health" or "first line" or "family healthcare") or ((family or general* or group) NEAR/1 (doctor* or physician* or pract* or medicine or nurs*)) or (rural NEAR/2 (physician* or practice or service* or hospital*)) or generalist* or (ambulatory NEAR/1 (care or clinic* or service*)) or (health NEAR/2 (center* or centre*)) or consult* or (visit* NEAR/2 (clinic* or care or outpatient* or office*)) or (community NEAR/2 (care or worker* or service* or nurs* or pract*)) or (social NEAR/1 (worker* or service*)) or practitioner* or "clinical practice*" or pharmacist* or dietitian* or hospice* or ((home or domicil*) NEAR/2 (care or healthcare or nurs* or rehabilit* or service* or visiting or visit?)) or homecare* or (nurse NEAR/0 (rehabilitator* or clinician*)) or paramedic* or ((emergency or emergencies or trauma) NEAR/2 (department* or service* or outpatient* or ward* or room* or unit or units or center* or centre* or physician* or nurse*)) or "ED" or "family health" or midwi*:ti,ab,kw)<br>Indexes=SCI-EXPANDED, SSCI, A&HCI, ESCI Timespan=All years | #2 | 1 220 467 |
| Total result | | #1 AND #2 | #3 | 8 995 |

# Cinahl (2020-02-10)

| PICOSS | Concepts | Research strategy keywords | Research | # Results |
|---|---|---|---|---|
| Population | Artificial Intelligence (Controlled Vocabulary) | (MH "Artificial Intelligence+") OR (MH "Data Mining") | #1 | 18 211 |
| | Artificial Intelligence (Free text) | TI ( "artificial intelligence*" or "computational intelligence*" or "machine intelligence*" or "automated reasoning" or "bayesian network*" or "bayes network*" or "naive bayes" or "bayesian learning" or "computer heuristic*" or "computer reasoning" or "data mining" or "text mining" or "expert system*" or "fuzzy logic" or "fuzzy cognitive" or "knowledge representation*" or "knowledge acquisition*" or "machine learning" or "learning machine*" or "natural language processing*" or "neural network*" or "deep learning" or "support vector*" or "hidden markov model*" or "random forest*" or "random decision forest*" or "supervised learning" or "unsupervised learning" or "autoencoder*" or "Generative adversarial network*" or "reservoir computing" or "shallow learning" or "echo state network*" or "case-based reasoning" or "metaheuristic*" or "soft computing" or "approximate reasoning" or "evolutionary computing" or "genetic algorithm*" or "bio-inspired algorithm*" )<br><br>OR AB ( "artificial intelligence*" or "computational intelligence*" or "machine intelligence*" or "automated reasoning" or "bayesian network*" or "bayes network*" or "naive bayes" or "bayesian learning" or "computer heuristic*" or "computer reasoning" or "data mining" or "text mining" or "expert system*" or "fuzzy logic" or "fuzzy cognitive" or "knowledge representation*" or "knowledge acquisition*" or "machine learning" or "learning machine*" or "natural language processing*" or "neural network*" or "deep learning" or "support vector*" or "hidden markov model*" or "random forest*" or "random decision forest*" or "supervised learning" or "unsupervised learning" or "autoencoder*" or "Generative adversarial network*" or "reservoir computing" or "shallow learning" or "echo state network*" or "case-based reasoning" or "metaheuristic*" or "soft computing" or "approximate reasoning" or "evolutionary computing" or "genetic algorithm*" or "bio-inspired algorithm*" )<br><br>OR MW ( "artificial intelligence*" or "computational intelligence*" or "machine intelligence*" or "automated reasoning" or "bayesian network*" or "bayes network*" or "naive bayes" or "bayesian learning" or "computer heuristic*" or "computer reasoning" or "data mining" or "text mining" or "expert system*" or "fuzzy logic" or "fuzzy cognitive" or "knowledge representation*" or "knowledge acquisition*" or "machine learning" or "learning machine*" or "natural language processing*" or "neural network*" or "deep learning" or "support vector*" or "hidden markov model*" or "random forest*" or "random decision forest*" or "supervised learning" or "unsupervised learning" or "autoencoder*" or "Generative adversarial network*" or "reservoir computing" or "shallow learning" or "echo state network*" or "case-based reasoning" or "metaheuristic*" or "soft computing" or "approximate reasoning" or "evolutionary computing" or "genetic algorithm*" or "bio-inspired algorithm*" ) | #2 | 20 450 |

| PICOSS | Concepts | Research strategy keywords | Research | # Results |
|---|---|---|---|---|
| | Artificial Intelligence | #1 OR #2 | #3 | 28 200 |
| Settings | Community-Based Primary Health Care (Controlled vocabulary | (MH "Primary Health Care") OR (MH "Family Practice") OR (MH "Physicians, Family") OR (MH "Group Practice+") OR (MH "Ambulatory Care Facilities") OR (MH "Ambulatory Care Facilities+") OR (MH "Community Health Centers+") OR (MH "Community Health Services+") OR (MH "Community Health Workers") OR (MH "Rural Health Personnel") OR (MH "Rural Health Nursing") OR (MH "Rural Health Centers") OR (MH "Rural Health Services") OR (MH "Nurse Practitioners+") OR (MH "Community Medicine") OR (MH "Community Health Nursing+") OR (MH "Clinical Nurse Specialists") OR (MH "Nurse Midwives") OR (MH "Nurse Practitioners") OR (MH "Pharmacists") OR (MH "Home Health Care+") OR (MH "Home Nursing") OR (MH "Hospices") OR (MH "Office Visits") OR (MH "Home Visits") OR (MH "Emergency Service+") OR (MH "Physicians, Emergency") OR (MH "Pharmacy Technicians") OR (MH "Emergency Medical Services+") OR (MH "Family Health") | #4 | 631 548 |
| | Community-Based Primary Health Care (Free text) | TI ( (primary N2 care) or ("primary healthcare" or "primary health" or "first line" or "family healthcare") or ((family or general* or group) N1 (doctor* or physician* or pract* or medicine or nurs*)) or (rural N2 (physician* or practice or service* or hospital*)) or generalist* or (ambulatory N1 (care or clinic* or service*)) or (health N2 (center* or centre*)) or consult* or (visit* N2 (clinic* or care or outpatient* or office*)) or (community N2 (care or worker* or service* or nurs* or pract*)) or (social N1 (worker* or service*)) or practitioner* or "clinical practice*" or pharmacist* or dietitian* or hospice* or ((home or domicil*) N2 (care or healthcare or nurs* or rehabilit* or service* or visiting or visit?))or homecare* or (nurse N0 (rehabilitator* or clinician*)) or paramedic* or ((emergency or emergencies or trauma) N2 (department* or service* or outpatient* or ward* or room* or unit or units or center* or centre* or physician* or nurse*)) or "ED" or "family health" or midwi* )<br><br>OR AB ( (primary N2 care) or ("primary healthcare" or "primary health" or "first line" or "family healthcare") or ((family or general* or group) N1 (doctor* or physician* or pract* or medicine or nurs*)) or (rural N2 (physician* or practice or service* or hospital*)) or generalist* or (ambulatory N1 (care or clinic* or service*)) or (health N2 (center* or centre*)) or consult* or (visit* N2 (clinic* or care or outpatient* or office*)) or (community N2 (care or worker* or service* or nurs* or pract*)) or (social N1 (worker* or service*)) or practitioner* or "clinical practice*" or pharmacist* or dietitian* or hospice* or ((home or domicil*) N2 (care or healthcare or nurs* or rehabilit* or service* or visiting or visit?)) or homecare* or (nurse N0 (rehabilitator* or clinician*)) or paramedic* or ((emergency or emergencies or trauma) N2 (department* or | #5 | 784 417 |

| | | | | |
|---|---|---|---|---|
| | | service* or outpatient* or ward* or room* or unit or units or center* or centre* or physician* or nurse*)) or "ED" or "family health" or midwi*)<br><br>OR SU ( (primary N2 care) or ("primary healthcare" or "primary health" or "first line" or "family healthcare") or ((family or general* or group) N1 (doctor* or physician* or pract* or medicine or nurs*)) or (rural N2 (physician* or practice or service* or hospital*)) or generalist* or (ambulatory N1 (care or clinic* or service*)) or (health N2 (center* or centre*)) or consult* or (visit* N2 (clinic* or care or outpatient* or office*)) or (community N2 (care or worker* or service* or nurs* or pract*)) or (social N1 (worker* or service*)) or practitioner* or "clinical practice*" or pharmacist* or dietitian* or hospice* or ((home or domicil*) N2 (care or healthcare or nurs* or rehabilit* or service* or visiting or visit?)) or homecare* or (nurse N0 (rehabilitator* or clinician*)) or paramedic* or ((emergency or emergencies or trauma) N2 (department* or service* or outpatient* or ward* or room* or unit or units or center* or centre* or physician* or nurse*)) or "ED" or "family health" or midwi*) | | |
| | Primary Health Care | #4 OR #5 | #6 | 1 051 685 |
| Total result | | #3 AND #6 | #7 | 3 196 |
| | Conference | Not applicable | #8 | 3 196 |

# IEE Explore (2020-02-07)

Given the particularities of the IEE Explore database, the search strategy was broken down into several elements that were subsequently recombined in using the search history. In the Search Combinations table, the result given for each strategy combination is the one obtained after the conferences have been withdrawn. After removing the duplicates in Endnote, we arrive at a total of 728 unique references for this database.

| PICOSS | Concepts | Research strategy keywords | Research | # Results |
|---|---|---|---|---|
| Population | Artificial Intelligence (Free text) | "artificial intelligence" OR "artificial intelligences" OR "computational intelligence" OR "computational intelligences" OR "machine intelligence" OR "machine intelligences" OR "automated reasoning" | #1 | 253 298 |
| | | "bayesian network" OR "bayesian networks" OR "bayes network" OR "bayes networks" OR "naive bayes" OR "bayesian learning" OR "computer heuristic" | #2 | 10 859 |
| | | "computer heuristics" OR "computer reasoning" OR "data mining" OR "text mining" OR "expert system" OR "expert systems" OR "fuzzy logic" | #3 | 151 681 |
| | | "fuzzy cognitive" OR "knowledge representation*" OR "knowledge representations" OR "knowledge acquisition" OR "knowledge acquisitions" OR "machine learning" OR "learning machine*" | #4 | 89 807 |
| | | "learning machines" OR "natural language processing" OR "natural language processings" OR "neural network*" OR "neural networks" OR "deep learning" OR "support vector" | #5 | 253 784 |
| | | "support vectors" OR "hidden markov" OR "random forest" OR "random forests" OR "random decision forest" OR "random decision forests" OR "supervised learning" | #6 | 44 822 |
| | | "unsupervised learning" OR autoencoder OR "Generative adversarial network" OR "Generative adversarial networks" OR "reservoir computing" OR "shallow learning" | #7 | 14 667 |
| | | "echo state network" OR "echo state networks" OR "case-based reasoning" OR metaheuristic OR "soft computing" OR "approximate reasoning" | #8 | 15 170 |
| | | "evolutionary computing" OR "genetic algorithm" OR "genetic algorithms" OR "bio-inspired algorithm" OR "bio-inspired algorithms" | #9 | 60 347 |

| PICOSS | Concepts | Research strategy keywords | Research | # Results |
|---|---|---|---|---|
| Settings | Community-Based Primary Health Care (Free text) | "primary care" OR "primary health care" OR "primary healthcare" OR "primary health" OR "first line" OR "family healthcare" OR generalist OR practitioner | #10 | **20 461** |
| | | "family doctor" OR "family doctors" OR "family physician" OR "family physicians" OR "family practice" OR "family practices" OR "family medicine" | #11 | **163** |
| | | "family medicines" OR "family nurse" OR "family nurses" OR "family nursing" OR "generalist doctor" OR "generalist doctors" OR "general doctor" | #12 | **9** |
| | | "general doctors" OR "general physician" OR "general physicians" OR "generalist physician" OR "generalist physicians" OR "general practice" OR "general practices" | #13 | **173** |
| | | "general medicine" OR "general medicines" OR "generalist medicine" OR "generalist medicines" OR "general nurse" OR "general nurses" OR "generalist nurse" | #14 | **33** |
| | | "generalist nurses" OR "general nursing" OR "generalist nursing" OR "doctor group" OR "doctors groups" OR "physician group" OR "physician groups" | #15 | **4** |
| | | "group practice" OR "group practices" OR "group medicine" OR "rural physician" OR "rural physicians" OR "rural practice" OR "rural practices" | #16 | **38** |
| | | "rural service" OR "rural services" OR "ambulatory care" OR "ambulatory health care" OR "ambulatory healthcare" OR "ambulatory clinic" OR "ambulatory clinics" | #17 | **121** |
| | | "health center" OR "health centers" OR "health centre" OR "health centres" OR "healthcare center" OR "healthcare centers" OR "healthcare centre" | #18 | **693** |
| | | "healthcare centres" OR "health care center" OR "health care centers" OR "health care centre" OR "health care centres" OR consultation | #19 | **1785** |
| | | "visit the clinic" OR "clinic visit" OR "clinic visits" OR "care visit" OR "care visits" OR "outpatient visit" OR "outpatient visits" | #20 | **62** |

| | | | | |
|---|---|---|---|---|
| | | "office visit" OR "office visits" OR "visit the office" OR "community care" OR "community health care" OR "community healthcare" | #21 | **141** |
| | | "community worker" OR "community workers" OR "community service" OR "community services" OR "community health service" OR "community health services" | #22 | **432** |
| | | "community mental health service" OR "community mental health services" OR "community nurse" OR "community nurses" OR "community nursing" | #23 | **12** |
| | | "community practice" OR "community practices" OR "social worker" OR "social workers" OR "social service" OR "social services" OR "clinical practice" | #24 | **2 460** |
| | | "clinical practices" OR pharmacist OR dietitian OR hospice OR homecare* Or "home care" OR "home healthcare" OR "home nursing" OR "home rehabilitation" | #25 | **845** |
| | | "home service" OR "home services" OR "visiting at home" OR "visit at home" OR "home visit" OR "home visits" | #26 | **478** |
| | | "domicile care" OR "domicile healthcare" OR "domicile nursing" OR "domicile rehabilitation" OR "rehabilitation nurse" OR "rehabilitation nurses" OR "clinician nurse" | #27 | **0** |
| | | "clinician nurses" OR "nurse midwife" OR "nurse midwives" or paramedic OR "emergency department" OR "emergency departments" OR "emergency service" OR "emergency services" | #28 | **5 489** |
| | | "outpatient emergency" OR "emergency ward" OR "emergency wards" OR "emergency room" OR "emergency rooms" OR "emergency unit" OR "emergency units" | #29 | **222** |
| | | "emergency center" OR "emergency centers" OR "emergency centre" OR "emergency centres" OR "emergencies department" OR "emergencies departments" OR "emergencies service" | #30 | **56** |
| | | "emergencies services" OR "outpatient emergencies" OR "emergencies ward" OR "emergencies wards" OR "emergencies room" OR "emergencies rooms" OR "emergencies unit" | #31 | **2** |
| | | "emergencies units" OR "emergencies center" OR "emergencies centers" OR "emergencies centre" OR "emergencies centres" OR "family health" | #32 | **46** |
| | | Midwife or midwives or midwifery | #33 | **7** |

| Search combinations | | | | | | | | | |
|---|---|---|---|---|---|---|---|---|---|
| **Search** | **Results** | **Search** | **Results** | **Search** | **Results** | **Search** | **Results** | **Search** | **Results** |
| #1 AND #10 | **428** | #1 AND #25 | **20** | #2 AND #16 | **0** | #2 AND #31 | **0** | #3 AND #22 | **0** |
| #1 AND #11 | **0** | #1 AND #26 | **6** | #2 AND #17 | **0** | #2 AND #32 | **0** | #3 AND #23 | **1** |
| #1 AND #12 | **0** | #1 AND #27 | **0** | #2 AND #18 | **1** | #2 AND #33 | **0** | #3 AND #24 | **30** |
| #1 AND #13 | **7** | #1 AND #28 | **0** | #2 AND #19 | **1** | #3 AND #10 | **137** | #3 AND #25 | **4** |
| #1 AND #14 | **3** | #1 AND #29 | **37** | #2 AND #20 | **1** | #3 AND #11 | **0** | #3 AND #26 | **2** |
| #1 AND #15 | **0** | #1 AND #30 | **4** | #2 AND #21 | **0** | #3 AND #12 | **0** | #3 AND #27 | **0** |
| #1 AND #16 | **0** | #1 AND #31 | **1** | #2 AND #22 | **0** | #3 AND #13 | **2** | #3 AND #28 | **23** |
| #1 AND #17 | **1** | #1 AND #32 | **1** | #2 AND #23 | **0** | #3 AND #14 | **0** | #3 AND #29 | **3** |
| #1 AND #18 | **8** | #1 AND #33 | **0** | #2 AND #24 | **0** | #3 AND #15 | **0** | #3 AND #30 | **0** |
| #1 AND #19 | **31** | #2 AND #10 | **7** | #2 AND #25 | **2** | #3 AND #16 | **1** | #3 AND #31 | **0** |
| #1 AND #20 | **0** | #2 AND #11 | **0** | #2 AND #26 | **0** | #3 AND #17 | **0** | #3 AND #32 | **1** |
| #1 AND #21 | **1** | #2 AND #12 | **0** | #2 AND #27 | **0** | #3 AND #18 | **6** | #3 AND #33 | **0** |
| #1 AND #22 | **6** | #2 AND #13 | **0** | #2 AND #28 | **2** | #3 AND #19 | **30** | #4 AND #10 | **144** |
| #1 AND #23 | **0** | #2 AND #14 | **0** | #2 AND #29 | **0** | #3 AND #20 | **2** | #4 AND #11 | **0** |
| #1 AND #24 | **82** | #2 AND #15 | **0** | #2 AND #30 | **0** | #3 AND #21 | **0** | #4 AND #12 | **0** |

| Search | Results | Search | Results | Search | Results | Search | Results | Search | Results |
|---|---|---|---|---|---|---|---|---|---|
| #4 AND #13 | **2** | | | | | | | | |
| #4 AND #14 | **0** | #4 AND #29 | **3** | #5 AND #20 | **1** | #6 AND #11 | **0** | #6 AND #26 | **1** |
| #4 AND #15 | **0** | #4 AND #30 | **0** | #5 AND #21 | **1** | #6 AND #12 | **0** | #6 AND #27 | **0** |
| #4 AND #16 | **0** | #4 AND #31 | **0** | #5 AND #22 | **5** | #6 AND #13 | **0** | #6 AND #28 | **2** |
| #4 AND #17 | **0** | #4 AND #32 | **0** | #5 AND #23 | **0** | #6 AND #14 | **0** | #6 AND #29 | **1** |
| #4 AND #18 | **9** | #4 AND #33 | **0** | #5 AND #24 | **63** | #6 AND #15 | **0** | #6 AND #30 | **0** |
| #4 AND #19 | **8** | #5 AND #10 | **207** | #5 AND #25 | **18** | #6 AND #16 | **0** | #6 AND #31 | **0** |
| #4 AND #20 | **0** | #5 AND #11 | **2** | #5 AND #26 | **8** | #6 AND #17 | **0** | #6 AND #32 | **0** |
| #4 AND #21 | **1** | #5 AND #12 | **0** | #5 AND #27 | **0** | #6 AND #18 | **4** | #6 AND #33 | **0** |
| #4 AND #22 | **2** | #5 AND #13 | **7** | #5 AND #28 | **20** | #6 AND #19 | **1** | #7 AND #10 | **15** |
| #4 AND #23 | **0** | #5 AND #14 | **1** | #5 AND #29 | **5** | #6 AND #20 | **0** | #7 AND #11 | **0** |
| #4 AND #24 | **43** | #5 AND #15 | **0** | #5 AND #30 | **1** | #6 AND #21 | **0** | #7 AND #12 | **0** |
| #4 AND #25 | **10** | #5 AND #16 | **0** | #5 AND #31 | **0** | #6 AND #22 | **0** | #7 AND #13 | **0** |
| #4 AND #26 | **2** | #5 AND #17 | **0** | #5 AND #32 | **0** | #6 AND #23 | **0** | #7 AND #14 | **0** |
| #4 AND #27 | **0** | #5 AND #18 | **9** | #5 AND #33 | **0** | #6 AND #24 | **13** | #7 AND #15 | **0** |
| #4 AND #28 | **15** | #5 AND #19 | **13** | #6 AND #10 | **36** | #6 AND #25 | **6** | #7 AND #16 | **0** |

| Search | Results | Search | Results | Search | Results | Search | Results | Search | Results |
|---|---|---|---|---|---|---|---|---|---|
| #7 AND #17 | **0** | #7 AND #32 | **0** | #8 AND #23 | **0** | #9 AND #14 | **0** | #9 AND #29 | **0** |
| #7 AND #18 | **0** | #7 AND #33 | **0** | #8 AND #24 | **2** | #9 AND #15 | **0** | #9 AND #30 | **0** |
| #7 AND #19 | **3** | #8 AND #10 | **15** | #8 AND #25 | **0** | #9 AND #16 | **0** | #9 AND #31 | **0** |
| #7 AND #20 | **0** | #8 AND #11 | **0** | #8 AND #26 | **0** | #9 AND #17 | **0** | #9 AND #32 | **0** |
| #7 AND #21 | **0** | #8 AND #12 | **0** | #8 AND #27 | **0** | #9 AND #18 | **0** | #9 AND #33 | **0** |
| #7 AND #22 | **0** | #8 AND #13 | **0** | #8 AND #28 | **0** | #9 AND #19 | **1** | | |
| #7 AND #23 | **0** | #8 AND #14 | **0** | #8 AND #29 | **0** | #9 AND #20 | **1** | | |
| #7 AND #24 | **5** | #8 AND #15 | **0** | #8 AND #30 | **0** | #9 AND #21 | **0** | | |
| #7 AND #25 | **0** | #8 AND #16 | **0** | #8 AND #31 | **0** | #9 AND #22 | **1** | | |
| #7 AND #26 | **0** | #8 AND #17 | **0** | #8 AND #32 | **0** | #9 AND #23 | **0** | | |
| #7 AND #27 | **0** | #8 AND #18 | **1** | #8 AND #33 | **0** | #9 AND #24 | **6** | | |
| #7 AND #28 | **0** | #8 AND #19 | **0** | #9 AND #10 | **26** | #9 AND #25 | **0** | | |
| #7 AND #29 | **0** | #8 AND #20 | **0** | #9 AND #11 | **0** | #9 AND #26 | **1** | | |
| #7 AND #30 | **0** | #8 AND #21 | **0** | #9 AND #12 | **0** | #9 AND #27 | **0** | | |
| #7 AND #31 | **0** | #8 AND #22 | **0** | #9 AND #13 | **0** | #9 AND #28 | **2** | | |

## Science Direct (2020-02-14)

Given the particularities of the Science Direct database, the search strategy was broken down into several elements that were subsequently recombined. In the « Title, abstract or author-specified keywords », two parts of the search strategy was combined by a AND. In the Search Combinations table, the result given for each strategy combination is the one obtained after the conferences have been withdrawn. After removing the duplicates in Endnote, we arrive at a total of 3100 unique references for this database.

| Search Combinations | | | | | |
|---|---|---|---|---|---|
| ("artificial intelligence" OR "computational intelligence" OR "machine intelligence" OR "automated reasoning" OR "bayesian network" OR "bayes network" OR "naive bayes" OR "bayesian learning") AND … | | | | | |
| CBHC terms | Results | CBHC terms | Results | CBHC terms | Results |
| "primary care" | 9 | "group medicine" | 0 | "visit the office" | 0 |
| "primary healthcare" | 2 | "rural practice" | 0 | "community care" | 1 |
| "primary health" | 0 | "health center" | 3 | "community health" | 1 |
| "first line" | 11 | "healthcare center" | 1 | "community healthcare" | 0 |
| "family healthcare" | 0 | "health care center" | 0 | "community worker" | 0 |
| generalist | 3 | consultation | 49 | "community service" | 0 |
| practitioner | 158 | "visit the clinic" | 0 | "community mental health service" | 0 |
| "family practice" | 2 | "clinic visit" | 0 | "community practice" | 0 |
| "family medicine" | 0 | "care visit" | 0 | "social worker" | 0 |
| "general practice" | 3 | "outpatient visit" | 2 | "social service" | 5 |
| "general medicine" | 3 | "office visit" | 0 | "clinical practice" | 116 |
| "group practice" | 0 | "visit the office" | 0 | pharmacist | 3 |

| Search Combinations | | | | | |
|---|---|---|---|---|---|
| ("artificial intelligence" OR "computational intelligence" OR "machine intelligence" OR "automated reasoning" OR "bayesian network" OR "bayes network" OR "naive bayes" OR "bayesian learning") AND … | | | | | |
| CBHC terms | Results | CBHC terms | Results | CBHC terms | Results |
| dietitian | 3 | "home service" | 2 | "domicile rehabilitation" | 0 |
| hospice | 1 | "visiting at home" | 0 | nursing | 26 |
| homecare | 2 | "visit at home" | 0 | nurse | 17 |
| "home care" | 2 | "home visit" | 0 | paramedic | 1 |
| "home healthcare" | 0 | "domicile care" | 0 | emergency | 146 |
| "home rehabilitation" | 0 | "domicile healthcare" | 0 | "family health" | 0 |
| midwife | 0 | midwives | 0 | midwifery | 0 |

| Search Combinations | | | | | |
|---|---|---|---|---|---|
| ("computer heuristic" OR "computer reasoning" OR "data mining" OR "text mining" OR "expert system" OR "fuzzy logic" OR "fuzzy cognitive" OR "knowledge representation") AND… | | | | | |
| CBHC terms | Results | CBHC terms | Results | CBHC terms | Results |
| "primary care" | 12 | "group medicine" | 0 | "visit the office" | 0 |
| "primary healthcare" | 0 | "rural practice" | 0 | "community care" | 0 |
| "primary health" | 8 | "health center" | 9 | "community health" | 3 |
| "first line" | 8 | "healthcare center" | 3 | "community healthcare" | 0 |
| "family healthcare" | 1 | "health care center" | 6 | "community worker" | 0 |
| generalist | 5 | consultation | 167 | "community service" | 0 |
| practitioner | 299 | "visit the clinic" | 0 | "community mental health service" | 0 |
| "family practice" | 1 | "clinic visit" | 1 | "community practice" | 0 |
| "family medicine" | 1 | "care visit" | 0 | "social worker" | 3 |
| "general practice" | 10 | "outpatient visit" | 4 | "social service" | 4 |
| "general medicine" | 2 | "office visit" | 2 | "clinical practice" | 93 |
| "group practice" | 0 | "visit the office" | 0 | pharmacist | 4 |

| Search Combinations | | | | | |
|---|---|---|---|---|---|
| ("computer heuristic" OR "computer reasoning" OR "data mining" OR "text mining" OR "expert system" OR "fuzzy logic" OR "fuzzy cognitive" OR "knowledge representation") AND… | | | | | |
| CBHC terms | Results | CBHC terms | Results | CBHC terms | Results |
| dietitian | 3 | "home service" | 0 | "domicile rehabilitation" | 0 |
| hospice | 0 | "visiting at home" | 0 | nursing | 48 |
| homecare | 3 | "visit at home" | 0 | nurse | 50 |
| "home care" | 6 | "home visit" | 0 | paramedic | 3 |
| "home healthcare" | 2 | "domicile care" | 0 | emergency | 192 |
| "home rehabilitation" | 0 | "domicile healthcare" | 0 | "family health" | 2 |
| Midwife | 0 | midwives | 0 | midwifery | 0 |

| Search Combinations | | | | | |
|---|---|---|---|---|---|
| ("knowledge acquisition" OR "machine learning" OR "learning machine" OR "natural language processing" OR "neural network" OR "deep learning" OR "support vector" OR "hidden markov") AND … | | | | | |
| CBHC terms | Results | CBHC terms | Results | CBHC terms | Results |
| "primary care" | 36 | "group medicine" | 0 | "visit the office" | 0 |
| "primary healthcare" | 6 | "rural practice" | 0 | "community care" | 2 |
| "primary health" | 10 | "health center" | 20 | "community health" | 10 |
| "first line" | 23 | "healthcare center" | 3 | "community healthcare" | 1 |
| "family healthcare" | 0 | "health care center" | 7 | "community worker" | 0 |
| generalist | 11 | consultation | 66 | "community service" | 1 |
| practitioner | 419 | "visit the clinic" | 1 | "community mental health service" | 0 |
| "family practice" | 3 | "clinic visit" | 3 | "community practice" | 0 |
| "family medicine" | 1 | "care visit" | 2 | "social worker" | 0 |
| "general practice" | 8 | "outpatient visit" | 6 | "social service" | 8 |
| "general medicine" | 2 | "office visit" | 1 | "clinical practice" | 363 |
| "group practice" | 2 | "visit the office" | 0 | pharmacist | 19 |

| Search Combinations | | | | | |
|---|---|---|---|---|---|
| ("knowledge acquisition" OR "machine learning" OR "learning machine" OR "natural language processing" OR "neural network" OR "deep learning" OR "support vector" OR "hidden markov") AND … | | | | | |
| CBHC terms | Results | CBHC terms | Results | CBHC terms | Results |
| dietitian | 5 | "home service" | 1 | "domicile rehabilitation" | 0 |
| hospice | 2 | "visiting at home" | 0 | nursing | 149 |
| homecare | 3 | "visit at home" | 0 | nurse | 122 |
| "home care" | 15 | "home visit" | 1 | paramedic | 4 |
| "home healthcare" | 2 | "domicile care" | 0 | emergency | 321 |
| "home rehabilitation" | 1 | "domicile healthcare" | 0 | "family health" | 1 |
| Midwife | 0 | midwives | 6 | midwifery | 9 |

| Search Combinations | | | | | |
|---|---|---|---|---|---|
| ("random forest" OR "random decision forest" OR "supervised learning" OR "unsupervised learning" OR autoencoder OR "Generative adversarial network" OR "reservoir computing" OR "shallow learning") AND … | | | | | |
| CBHC terms | Results | CBHC terms | Results | CBHC terms | Results |
| "primary care" | 36 | "group medicine" | 0 | "visit the office" | 0 |
| "primary healthcare" | 6 | "rural practice" | 0 | "community care" | 0 |
| "primary health" | 10 | "health center" | 2 | "community health" | 1 |
| "first line" | 7 | "healthcare center" | 1 | "community healthcare" | 0 |
| "family healthcare" | 0 | "health care center" | 0 | "community worker" | 0 |
| generalist | 4 | consultation | 5 | "community service" | 0 |
| practitioner | 49 | "visit the clinic" | 0 | "community mental health service" | 0 |
| "family practice" | 0 | "clinic visit" | 0 | "community practice" | 0 |
| "family medicine" | 0 | "care visit" | 0 | "social worker" | 0 |
| "general practice" | 1 | "outpatient visit" | 0 | "social service" | 2 |
| "general medicine" | 1 | "office visit" | 0 | "clinical practice" | 41 |
| "group practice" | 0 | "visit the office" | 0 | pharmacist | 2 |

| Search Combinations | | | | | |
|---|---|---|---|---|---|
| ("random forest" OR "random decision forest" OR "supervised learning" OR "unsupervised learning" OR autoencoder OR "Generative adversarial network" OR "reservoir computing" OR "shallow learning") AND … | | | | | |
| CBHC terms | Results | CBHC terms | Results | CBHC terms | Results |
| dietitian | 0 | "home service" | 0 | "domicile rehabilitation" | 0 |
| hospice | 0 | "visiting at home" | 0 | nursing | 4 |
| homecare | 0 | "visit at home" | 0 | nurse | 3 |
| "home care" | 2 | "home visit" | 1 | paramedic | 1 |
| "home healthcare" | 0 | "domicile care" | 0 | emergency | 38 |
| "home rehabilitation" | 1 | "domicile healthcare" | 0 | "family health" | 0 |
| Midwife | 0 | midwives | 2 | midwifery | 0 |

| Search Combinations | | | | | |
|---|---|---|---|---|---|
| ("echo state network" OR "case-based reasoning" OR "metaheuristic" OR "soft computing" OR "approximate reasoning" OR "evolutionary computing" OR "genetic algorithm" OR "bio-inspired algorithm") AND… | | | | | |
| CBHC terms | Results | CBHC terms | Results | CBHC terms | Results |
| "primary care" | 3 | "group medicine" | 0 | "visit the office" | 0 |
| "primary healthcare" | 0 | "rural practice" | 0 | "community care" | 1 |
| "primary health" | 0 | "health center" | 1 | "community health" | 1 |
| "first line" | 2 | "healthcare center" | 1 | "community healthcare" | 0 |
| "family healthcare" | 0 | "health care center" | 1 | "community worker" | 0 |
| generalist | 3 | consultation | 9 | "community service" | 0 |
| practitioner | 165 | "visit the clinic" | 0 | "community mental health service" | 0 |
| "family practice" | 1 | "clinic visit" | 0 | "community practice" | 0 |
| "family medicine" | 0 | "care visit" | 0 | "social worker" | 0 |
| "general practice" | 6 | "outpatient visit" | 1 | "social service" | 1 |
| "general medicine" | 0 | "office visit" | 0 | "clinical practice" | 24 |
| "group practice" | 0 | "visit the office" | 0 | pharmacist | 6 |

| Search Combinations | | | | | |
|---|---|---|---|---|---|
| ("echo state network" OR "case-based reasoning" OR "metaheuristic" OR "soft computing" OR "approximate reasoning" OR "evolutionary computing" OR "genetic algorithm" OR "bio-inspired algorithm") AND… | | | | | |
| CBHC terms | Results | CBHC terms | Results | CBHC terms | Results |
| dietitian | 1 | "home service" | 0 | "domicile rehabilitation" | 0 |
| hospice | 0 | "visiting at home" | 0 | nursing | 10 |
| homecare | 0 | "visit at home" | 0 | nurse | 29 |
| "home care" | 6 | "home visit" | 0 | paramedic | 0 |
| "home healthcare" | 3 | "domicile care" | 0 | emergency | 165 |
| "home rehabilitation" | 0 | "domicile healthcare" | 0 | "family health" | 0 |
| Midwife | 0 | midwives | 0 | midwifery | 0 |
