# Supplementary material for: Application of Artificial Intelligence in Community-Based Primary Health Care: Systematic Scoping Review and Critical Appraisal
Source: J Med Internet Res. 2021 Sep 3;23(9):e29839. doi: 10.2196/29839 (PMC8449300; doi:10.2196/29839)
Supplement: Multimedia Appendix 3 [file jmir_v23i9e29839_app3.pdf]

**Multimedia Appendix 3:** Timeline of artificial intelligence application in community based primary health care between 1990 and 2020.
ES – Expert Systems, KNN – K Nearest Neighbours, ANN – Artificial Neural Networks, SVM – Support Vector Machine, GA – Genetic Algorithms, RF – Random Forests, LASSO – Least Absolute Shrinkage & Selection Operator, NLP – Natural Language Processing, LDA – Linear Discriminant Analysis, CNN – Convolutional Neural Networks, LS-SVM – Least-Squares Support Vector Machine, CRF – Conditional Random Field, HDP – Hierarchical Dirichlet Process , REDEx – Regular Expression Discovery for Expression, RIPPER – Repeated, Incremental Pruning to Produce Error Reduction, UIMA – Unstructured Information Management Architecture, DSS – Decision Support System, CBR – Case-based Reasoning

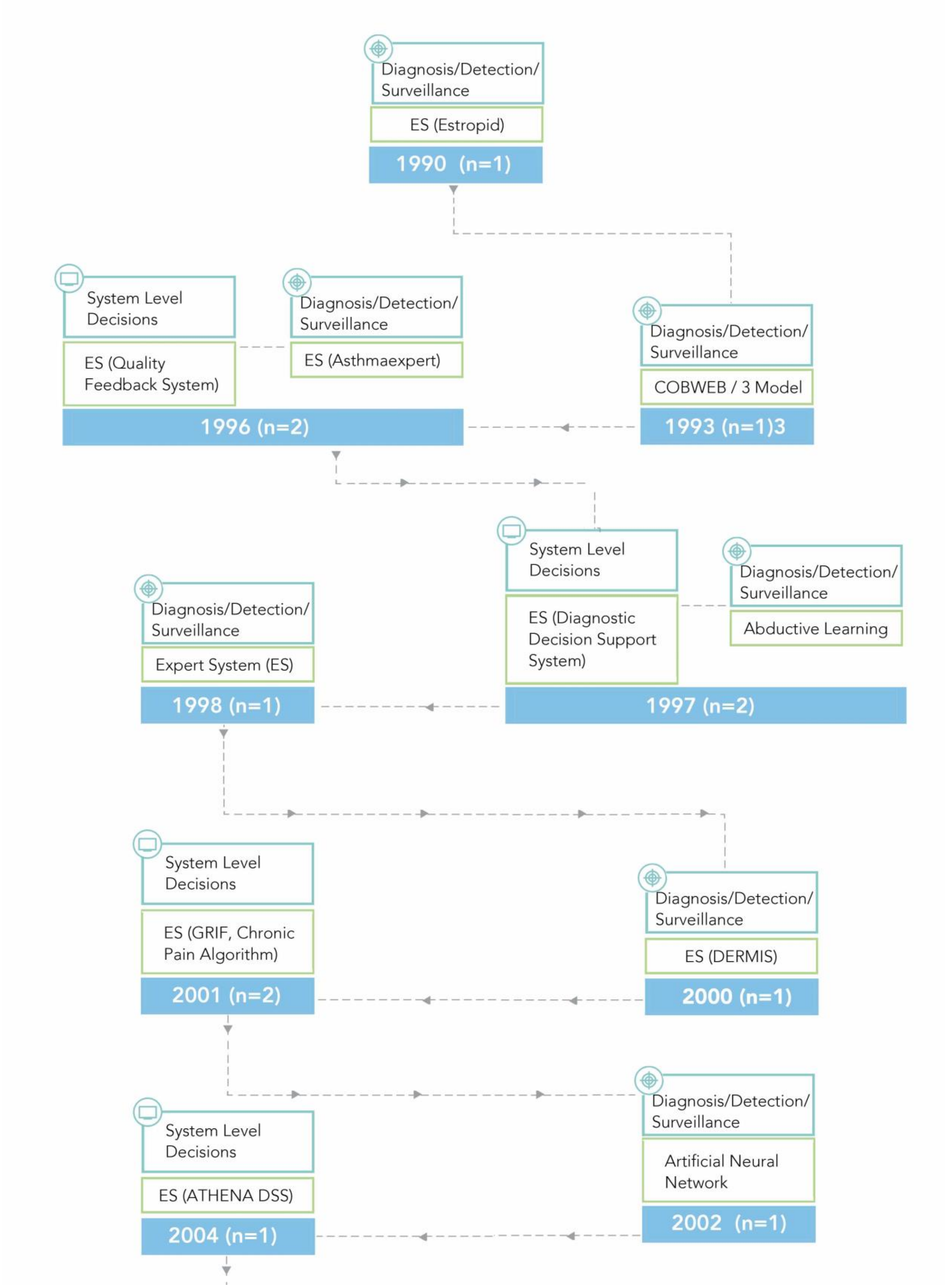
Diagnosis/Detection/ Surveillance
ES (Estropid)
1990 (n=1)
System Level Decisions
ES (Quality Feedback System)
Diagnosis/Detection/ Surveillance
ES (Asthmaexpert)
1996 (n=2)
Diagnosis/Detection/ Surveillance
COBWEB / 3 Model
1993 (n=1)3
System Level Decisions
ES (Diagnostic Decision Support System)
Diagnosis/Detection/ Surveillance
Abductive Learning
1997 (n=2)
Diagnosis/Detection/ Surveillance
Expert System (ES)
1998 (n=1)
System Level Decisions
ES (GRIF, Chronic Pain Algorithm)
2001 (n=2)
Diagnosis/Detection/ Surveillance
ES (DERMIS)
2000 (n=1)
System Level Decisions
ES (ATHENA DSS)
2004 (n=1)
Diagnosis/Detection/ Surveillance
Artificial Neural Network
2002 (n=1)

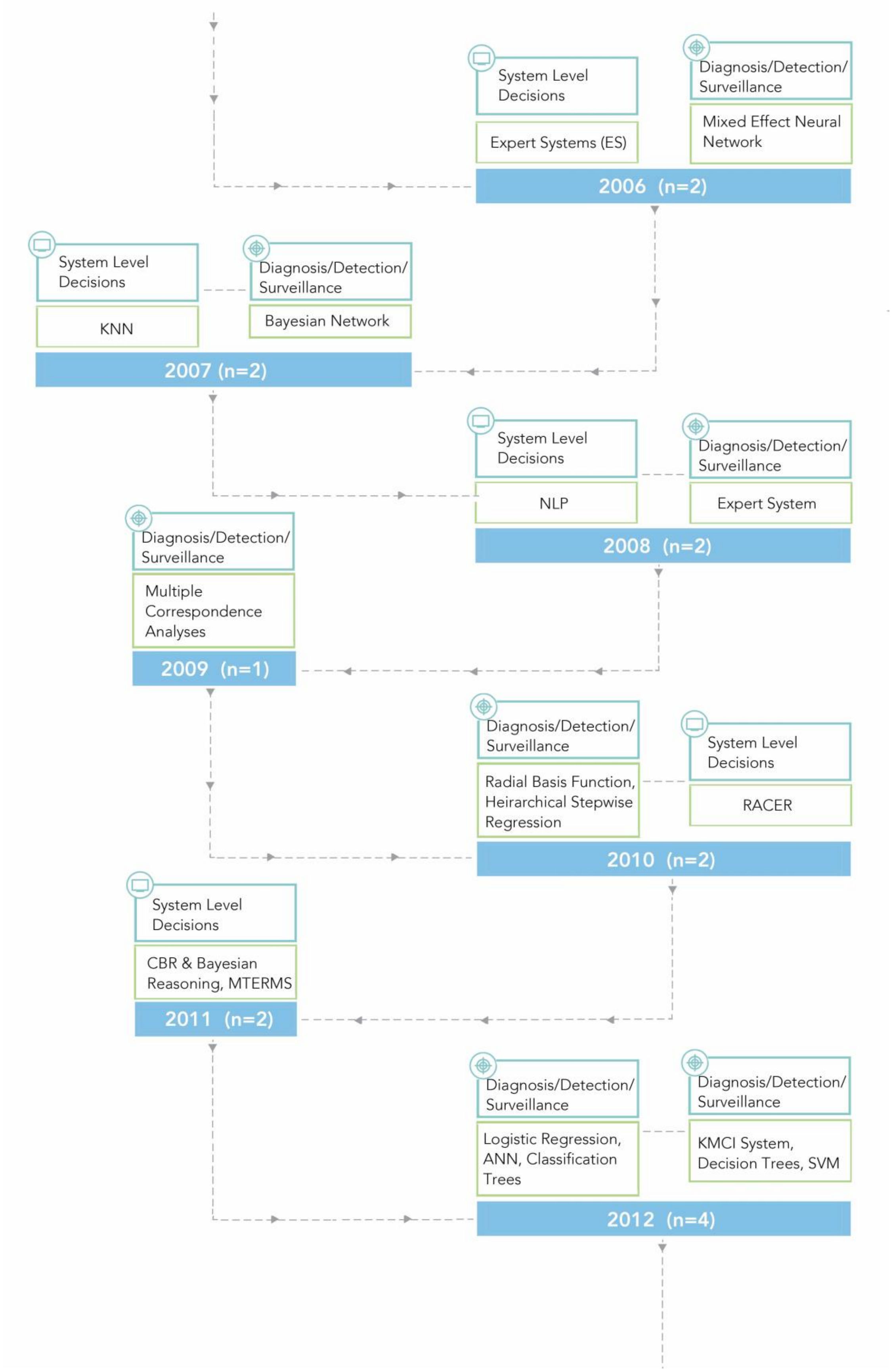
System Level Decisions
Expert Systems (ES)
Diagnosis/Detection/ Surveillance
Mixed Effect Neural Network
2006 (n=2)
System Level Decisions
KNN
Diagnosis/Detection/ Surveillance
Bayesian Network
2007 (n=2)
System Level Decisions
NLP
Diagnosis/Detection/ Surveillance
Expert System
2008 (n=2)
Diagnosis/Detection/ Surveillance
Multiple Correspondence Analyses
2009 (n=1)
Diagnosis/Detection/ Surveillance
Radial Basis Function, Heirarchical Stepwise Regression
System Level Decisions
RACER
2010 (n=2)
System Level Decisions
CBR & Bayesian Reasoning, MTERMS
2011 (n=2)
Diagnosis/Detection/ Surveillance
Logistic Regression, ANN, Classification Trees
Diagnosis/Detection/ Surveillance
KMCI System, Decision Trees, SVM
2012 (n=4)

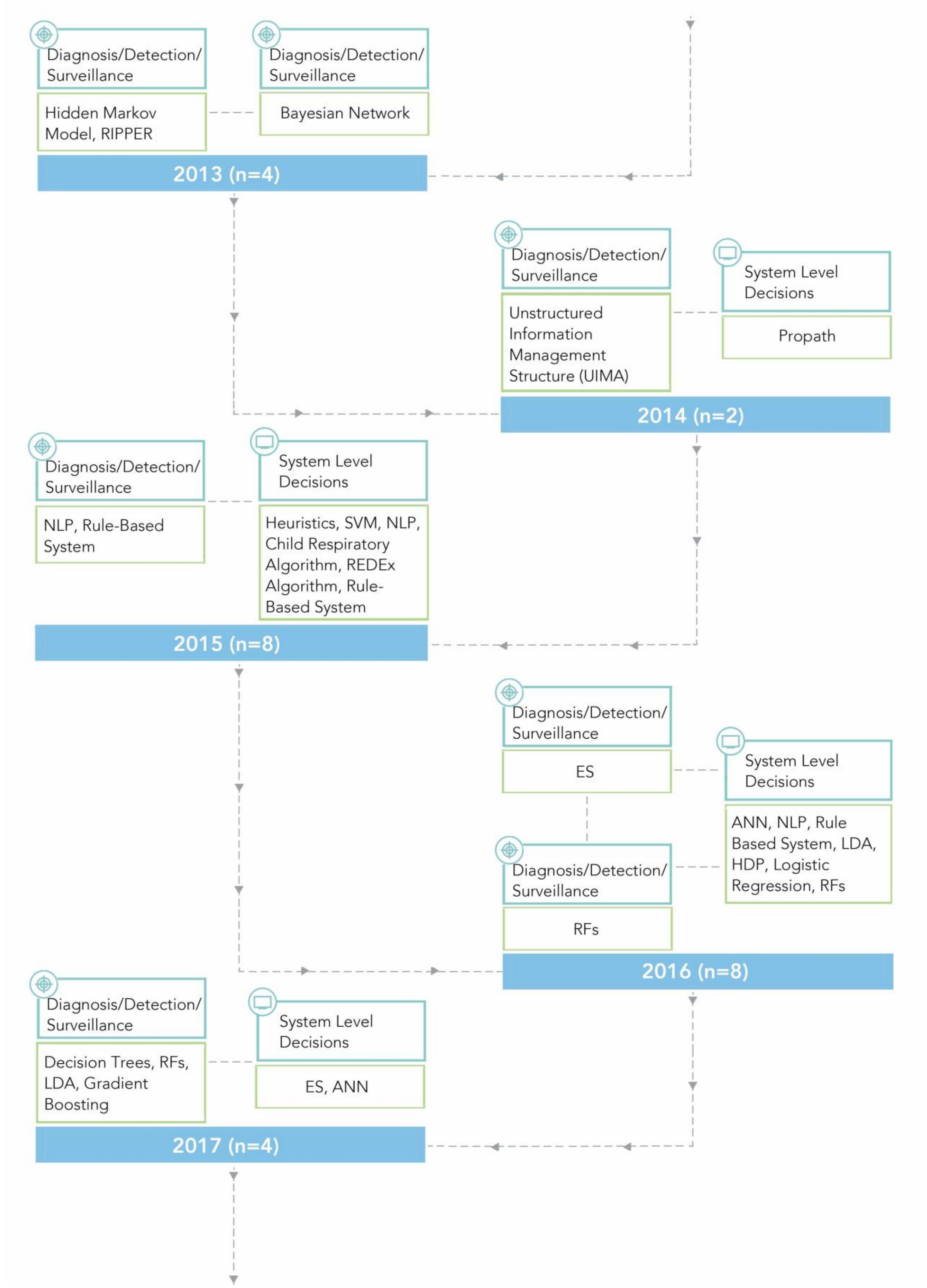
Diagnosis/Detection/ Surveillance
Hidden Markov Model, RIPPER
Diagnosis/Detection/ Surveillance
Bayesian Network
2013 (n=4)
Diagnosis/Detection/ Surveillance
Unstructured Information Management Structure (UIMA)
System Level Decisions
Propath
2014 (n=2)
Diagnosis/Detection/ Surveillance
NLP, Rule-Based System
System Level Decisions
Heuristics, SVM, NLP, Child Respiratory Algorithm, REDEx Algorithm, Rule-Based System
2015 (n=8)
Diagnosis/Detection/ Surveillance
ES
System Level Decisions
ANN, NLP, Rule Based System, LDA, HDP, Logistic Regression, RFs
Diagnosis/Detection/ Surveillance
RFs
2016 (n=8)
Diagnosis/Detection/ Surveillance
Decision Trees, RFs, LDA, Gradient Boosting
System Level Decisions
ES, ANN
2017 (n=4)

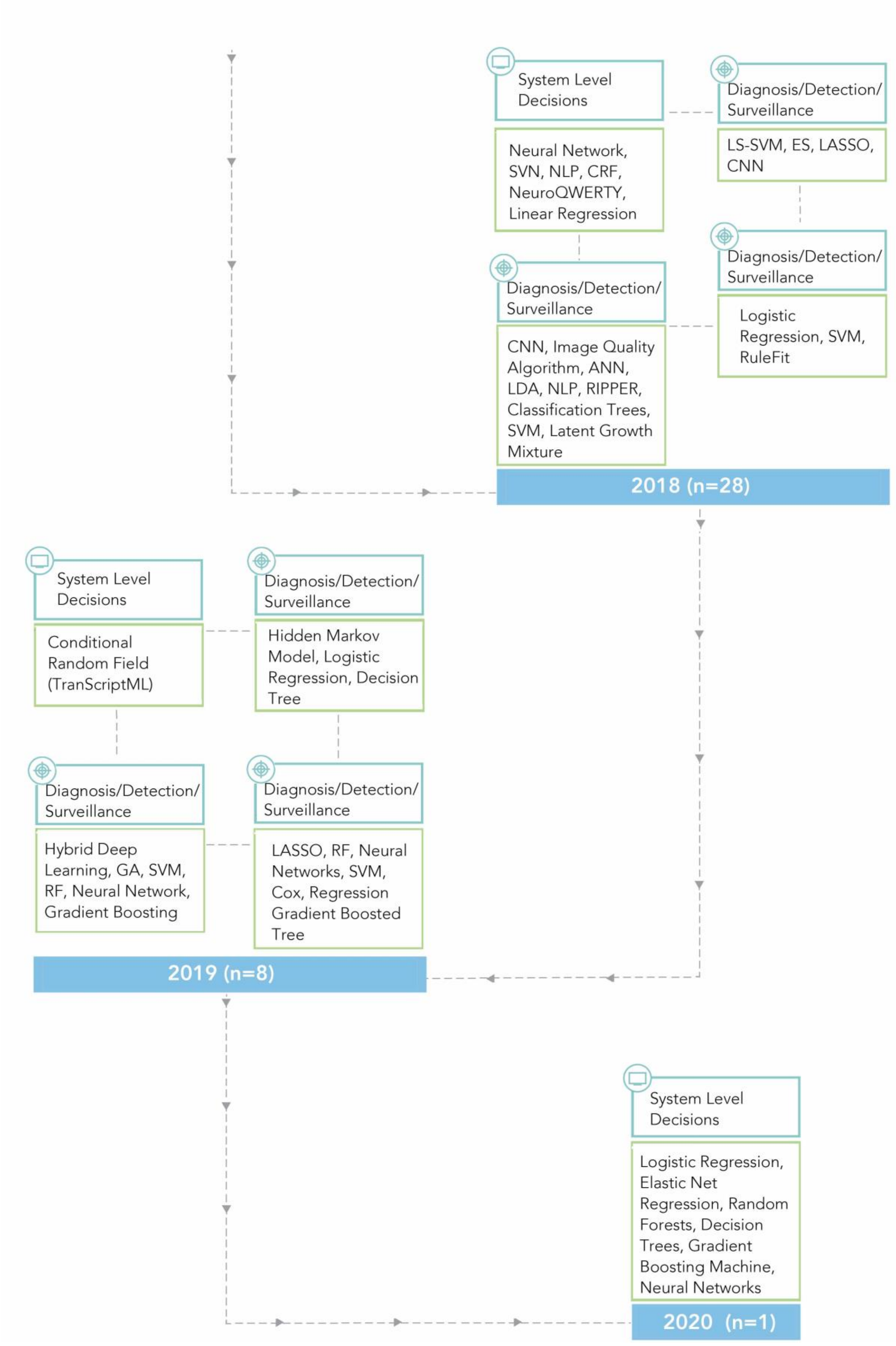
System Level Decisions
Neural Network, SVN, NLP, CRF, NeuroQWERTY, Linear Regression
Diagnosis/Detection/ Surveillance
LS-SVM, ES, LASSO, CNN
Diagnosis/Detection/ Surveillance
Logistic Regression, SVM, RuleFit
Diagnosis/Detection/ Surveillance
CNN, Image Quality Algorithm, ANN, LDA, NLP, RIPPER, Classification Trees, SVM, Latent Growth Mixture
2018 (n=28)
System Level Decisions
Conditional Random Field (TranScriptML)
Diagnosis/Detection/ Surveillance
Hidden Markov Model, Logistic Regression, Decision Tree
Diagnosis/Detection/ Surveillance
Hybrid Deep Learning, GA, SVM, RF, Neural Network, Gradient Boosting
Diagnosis/Detection/ Surveillance
LASSO, RF, Neural Networks, SVM, Cox, Regression Gradient Boosted Tree
2019 (n=8)
System Level Decisions
Logistic Regression, Elastic Net Regression, Random Forests, Decision Trees, Gradient Boosting Machine, Neural Networks
2020 (n=1)
