# Supplementary material for: Application of Artificial Intelligence in Community-Based Primary Health Care: Systematic Scoping Review and Critical Appraisal
Source: J Med Internet Res. 2021 Sep 3;23(9):e29839. doi: 10.2196/29839 (PMC8449300; doi:10.2196/29839)
Supplement: Multimedia Appendix 4 [file jmir_v23i9e29839_app4.pdf]

Application of Artificial Intelligence in Community-Based Primary Health Care: Systematic Scoping Review and Critical Appraisal

Multimedia Appendix 4: Some of the extracted data from the included studies

Author	Title	Publication Year	Objective	Input information	Details about the AI system	Performance	AI methods	Compared with other methods?	Eligible for PROBAST (Yes/No)
1. E. M. Moreno,M. J. A. Luján,M. T. Rusiñol,P. J. Fernández,P. N. Manrique,C. A. Triviño,M. P. Miquel,M. A. Rodríguez,M. J. G. Burguillos	Type 2 Diabetes Screening Test by Means of a Pulse Oximeter	2017	To screen for the presence of type 2 diabetes by means of the signal obtained from a pulse oximeter	Physiological parameters such as heart rate variability,	The system consists of a front end that extracts a set of features form the pulse oximeter signal. These features are based on physiological considerations and were the input for a machine-learning algorithm that determined the class of the input sample, i.e., whether the subject had diabetes or not	Mean ROC area 69.4% (median value 71.9% and range [75.4–61.1%]), with a specificity = 64% for a threshold that gave a sensitivity = 65%.	Random forests, gradient boosting, and linear discriminant analysis	Yes	Yes
2. S. Klausner,K. Entacher,S. Kranzer,A. Sönnichsen,M. Flamm,G. Fritsch	ProPath - A guideline-based software for the implementation into the medical environment	2014	To provide an overview of how acquired knowledge can be utilized to reduce the complexity of designing and implementing clinical pathways (ProPath),	Medical information	Details about the AI system not mentioned	Not reported	Expert system (ProPath)	No	No
3. G. Wang,Z. Deng,K. S. Choi	Tackling Missing Data in Community Health Studies Using Additive LS-SVM Classifier	2018	To predicting quality of life in community based primary health care.	Patients' demographics, socioeconomic status, health history; outcomes of health assessments; and quality of life measured via a standardized questionnaire of the World Health Organization	A new model introduced to deal with missing data. The new model applied to predict quality of life of community dwelling elderly.	Prediction accuracy 0.7418	Additive least square support vector machine (LS-SVM) Classifier	Yes	No
4. Safarova, Ms,Liu, H,Kullo, Ij	Rapid identification of familial hypercholesterolemia from electronic health records: the SEARCH study	2016	To develop an ePhenotyping algorithm for rapid identification of Family history in electronic health records (EHRs) and deploy it in the Screening Employees And Residents in the Community for Hypercholesterolemia (SEARCH) study.	Family history, Personal history of premature coronary heart disease and of cerebrovascular or peripheral arterial disease, lipid levels, race, low-density lipoprotein cholesterol (LDL-C)	EHR-based phenotyping algorithm that included NLP and had reasonable accuracy in identifying FH case status was used	Not reported	ePhenotyping algorithm	No	No
5. Lin, J,Bruni, Fm,Fu, Z,Maloney, J,Bardina, L,Boner, Al,Gimenez, G,Sampson, Ha	A bioinformatics approach to identify patients with symptomatic peanut allergy using peptide microarray immunoassay	2012	To develop an allergy diagnostic method that could correctly predict symptomatic peanut allergy by using peptide microarray immunoassays and bioinformatic methods.	Specific IgE and IgG4 binding to 419 overlapping peptides (15 mers, 3 offset) covering the amino acid sequences of Ara h 1, Ara h 2, and Ara h 3 were measured by using a peptide microarray immunoassay.	Bioinformatic methods were applied for data analysis, combined with the results of a peptide microarray immunoassay	Not reported	Decision trees and support vector machine	Yes	Yes
6. Witteman, Cl,Renooij, S,Koele, P	Medicine in words and numbers: a cross-sectional survey comparing probability assessment scales	2007	To compare assessments obtained with different types of response scale.	General practitioners gave assessments on and preferences for three different probability response scales: a numerical scale, a scale with only verbal labels, and a combined verbal- numerical scale designed by authors.	Standard analyses of variance were performed.	Not reported	Bayesian network	No	No

7. Tran, T. T.,Fang, T. Y.,Pham, V. T.,Lin, C.,Wang, P. C.,Lo, M. T.	Development of an Automatic Diagnostic Algorithm for Pediatric Otitis Media	2018	To develop an automatic diagnostic algorithm for pediatric Otitis Media (OM).	Otoscopic images	For the OM image classification system, the image database is randomly partitioned into the test and train subsets. Of each image in the train and test sets, the desired eardrum image region is first segmented, then multiple image features such as color, and shape are extracted. The multitask joint sparse representation-based classification to combine different features of the OM image is used for classification.	Classification accuracy as high as 91.41%.	Image based classification	Yes	Yes
8. Zheng, C.,Luo, Y.,Mercado, C.,Sy, L.,Jacobsen, S. J.,Ackerson, B.,Lewin, B.,Tseng, H. F.	Using natural language processing for identification of herpes zoster ophthalmicus cases to support population-based study	2018	1) To assess whether herpes zoster ophthalmicus can be identified from the clinical notes using natural language processing (NLP). 2) To investigate the epidemiology of HZO among HZ population based on the developed approach.	Patient characteristics, medical encounters in the 6 months before the HZ episode and co-morbidities	An NLP-based algorithm was developed and validated with the manually curated validation data set. The algorithm was applied on over 1 million clinical notes associated with the study population. HZO versus non-HZO cases were compared by age, sex, race and co-morbidities.	NLP algorithm achieved 95.6% sensitivity and 99.3% specificity	NLP algorithm (details not mentioned)	No	Yes
9. Gannod, G. C.,Abbott, K. M.,Van Haitsma, K.,Martindale, N.,Heppner, A.	A Machine Learning Recommender System to Tailor Preference Assessments to Enhance Person-Centered Care Among Nursing Home Residents	2018	To develop a machine learning recommender system to identify additional Preferences for Everyday Living Inventory (PELI-NH) items that may be important to specific residents.	Sample of NH resident responses to the PELI-NH	based on the concept of collaborative filtering whereby insights and predictions (e.g., filters) are created using the interests and preferences of many users. The algorithm identifies multiple sets of "you might also like" patterns called association rules, based upon responses	High precision of 80.2% and 79.2%	Expert system (Apriori and logistic regression model)	No	No
10. Lau, K.,Wilkinson, J.,Moorthy, R.	A web-based prediction score for head and neck cancer referrals	2018	To develop a scoring system that determines the risk of head and neck cancer and that can be used to aid general practitioner referrals.	The retrospective data collected from two different hospitals in different time periods. The data included patients' demographics, risk factors and relevant investigations (i.e., patient's ethnicity, age, sex, presenting signs or symptoms, smoking status, alcohol consumption, any other specific risk factors, any imaging done, any histology results and final outcome).	Logistic regression was used only. No clarification on any other used machine learning methods were provided.	Sensitivity of 31% and specificity of 92%.	Logistic regression	No	Yes

11. Jarvik, J. G.,Gold, L. S.,Tan, K.,Friedly, J. L.,Nedeljkovic, S. S.,Comstock, B. A.,Deyo, R. A.,Turner, J. A.,Bresnahan, B. W.,Rundell, S. D.,James, K. T.,Nerenz, D. R.,Avins, A. L.,Bauer, Z.,Kessler, L.,Heagerty, P. J.	Long-term outcomes of a large, prospective observational cohort of older adults with back pain	2018	1) To report 2-year outcomes of older adults for back pain in primary care setting. 2) To examine the relative importance of patient factors versus medical interventions in predicting 2-year disability and pain.	Patient self-report age, gender, race, ethnicity, education, employment status, marital status, and smoking status, the duration of current episode of back or leg pain and whether the patient had involvement with an attorney with a back-related claim or lawsuit. Patients also rated their confidence that their back or leg pain would be completely gone or much better in 3 months on a scale from 0 to 10. Patient-reported outcome measures (at 3, 6, and 12 months, and 2 years after enrollment) related to Pain-related characteristics, Psychological distress, Health-related quality of life, Falls, Body mass index, Quan comorbidity score, Baseline diagnosis, Number of relative value units, Spine-related interventions, Opioid prescriptions, and Days from index visit to consent were also collected and used.	Predictive model of least absolute shrinkage and selection operator (LASSO) regression method were used.	Regression model parameters explained 40% of the variation	LASSO	No	Yes
12. Chaudhry, A. P.,Afzal, N.,Abidian, M. M.,Mallipeddi, V. P.,Elayavilli, R. K.,Scott, C. G.,Kullo, I. J.,Wennberg, P. W.,Pankratz, J. J.,Liu, H.,Chaudhry, R.,Arruda-Olson, A. M.	Innovative Informatics Approaches for Peripheral Artery Disease: Current State and Provider Survey of Strategies for Improving Guideline-Based Care	2018	To quantify compliance with guideline recommendations for secondary prevention in peripheral artery disease (PAD) using natural language processing (NLP) tools deployed to an electronic health record (EHR) and investigate provider opinions regarding clinical decision support (CDS) to promote improved implementation of these strategies.	Narrative clinical notes of an EHR of patients seen in consultation for PAD cases	Natural language processing was used for automated identification of moderate to severe PAD cases. Subsequently, a provider survey was used to assess provider knowledge regarding PAD clinical practice guidelines, comfort in recommending secondary prevention strategies, and potential role for CDS.	the NLP algorithm had a PPV of 97% and sensitivity of 99% for automated PAD case identification.	NLP algorithm (details not mentioned)	No	No
13. Thabtah, F.,Kamalov, F.,Rajab, K.	A new computational intelligence approach to detect autistic features for autism screening	2018	To identify fewer, albeit influential, features in common ASD screening methods in order to achieve efficient screening as demands on evaluating the items' influences on ASD within existing tools is urgent.	Variable is an item/question in the screening method, and the class is whether an individual has ASD traits.	Variable Analysis considers feature-to-class correlations and reduces feature-to- feature correlations.	Not reported	Variable analysis, RIPPER, C4.5	Yes	Yes

14. Braido, F.,Santus, P.,Corsico, A. G.,Di Marco, F.,Melioli, G.,Scichilone, N.,Solidoro, P.	Chronic obstructive lung disease "expert system": validation of a predictive tool for assisting diagnosis	2018	To develop and validate an expert system that aimed to support the diagnosis of chronic obstructive lung disease.	An expert panel pilot validation on 60 cases and a clinical validation on 241 cases were performed. Age, sex, presence and characteristics of chronic cough, sputum and dyspnea, environmental exposure (smoke and/or known allergen sensitizations) and available diagnostic tests (chest X-ray, pre- and post-bronchodilator spirometry) were considered.	The expert system's code was written using WebFlex (an advanced knowledge specification language). The expert system is based on frame rules (representing the knowledge base) driving the system itself and on forms for input/output.	Accuracy of 97.5%,	Expert system	No	Yes
15. Du, L.,Xia, C.,Deng, Z.,Lu, G.,Xia, S.,Ma, J.	A machine learning based approach to identify protected health information in Chinese clinical text	2018	1) To describe the current process for protected health information in China, (2) To propose a machine learning based approach to identify PHI in Chinese clinical text(3) To validate the effectiveness of the machine learning algorithm for de-identification in Chinese clinical text.	Discharge summaries from regional health centers	Built a conditional random fields (CRF) model to identify protected health information in clinical text, and then used the regular expressions to optimize the recognition results of the PHI categories with fewer samples.	F-measure of 0.9878	Conditional random field	Yes	No
16. Erickson, J.,Abbott, K.,Susienka, L.	Automatic address validation and health record review to identify homeless Social Security disability applicants	2018	To explore address validation and automatic annotation of electronic health records to improve identification of homeless patients.	Patient's street address, geographic ID, block number, ZIP code, city, or county, medical records and disability applications.	Trained five random forests on dataset: The first random forest used all predictor variables, and the remaining random forests used only subsets of predictor variables, to help characterize relative contributions of classes of variables to primary model.	ROC was 0.942.	NLP (details not mentioned), Random forest	Yes	No
17. Turner, N. M.,MacRae, J.,Nowlan, M. L.,McBain, L.,Stubbe, M. H.,Dowell, A.	Quantifying the incidence and burden of herpes zoster in New Zealand general practice: a retrospective cohort study using a natural language processing software inference algorithm	2018	To investigate the incidence of primary care presentations for herpes zoster (zoster) in a representative New Zealand population 2) To evaluate the utilisation of primary healthcare services following zoster diagnosis.	Electronic medical records (EMRs)	A natural language processing software inference algorithm was used to identify general practice consultations for zoster by interrogating EMRs transactions routinely recorded	95% CI 47.6 to 49.6	Natural language processing algorithm (details not mentioned)	Yes	No
18. Luo, L.,Small, D.,Stewart, W. F.,Roy, J. A.	Methods for estimating kidney disease stage transition probabilities using electronic medical records	2013	To predict kidney disease stage transition rate using primary care electronic medical records.	Laboratory orders and results, medication orders, and inpatient and outpatient encounters, demographic variables (such as age and gender), and dates that the patients had dialysis, a kidney transplant, or died.	Developed an approach for estimation based on using hidden Markov models and simulation studies were used.	Not reported	Hidden Markov model	No	Yes

19. Lee, S. I.,Adans-Dester, C. P.,Grimaldi, M.,Dowling, A. V.,Horak, P. C.,Black-Schaffer, R. M.,Bonato, P.,Gwin, J. T.	Enabling Stroke Rehabilitation in Home and Community Settings: A Wearable Sensor-Based Approach for Upper-Limb Motor Training	2018	To detect goal-directed upper limb movements during the performance of ADL, so that timely feedback can be provided to encourage the use of the affected limb, 2) To assess the quality of motor performance during in-home rehabilitation exercises so that appropriate feedback can be generated to promote high-quality exercise.	Motor tasks, except for some passive movements	The performance of the classifier was assessed using the leave-one-subject-out cross validation technique. This technique evaluates the performance of an algorithm by selecting the data belonging to each subject (one at the time) as the test set and by training the algorithm using the data belonging to the remaining subjects.	C-statistic of 87.0% & 2, F -score of 84.3%	Logistic regression	No	No
20. Morales, D. R.,Flynn, R.,Zhang, J.,Trucco, E.,Quint, J. K.,Zutis, K.	External validation of ADO, DOSE, COTE and CODEX at predicting death in primary care patients with COPD using standard and machine learning approaches.	2018	To evaluate several existing risk scores for mortality prediction in Chronic obstructive pulmonary disease (COPD) among COPD population from primary care.	Symptoms, diagnoses, tests, referrals to secondary care and death. Around 60% of the patients included in the UK Clinical Practice Research Datalink have been linked to an administrative database containing information on all death registrations in England.	Used the logistic regression and LibSVM implementation of support vector machines (SVMs).	Not reported	Logistic regression, support vector model	No	Yes
21. Tou, H.,Yao, L.,Wei, Z.,Zhuang, X.,Zhang, B.	Automatic infection detection based on electronic medical records.	2018	To propose the use of machine learning approaches for automatic infection detection based on EMRs.	Five categories of information are utilized for prediction, including personal information, admission note, vital signs, diagnose test results and medical image diagnose.	Used sklearn for model implementation. To explore the effectiveness of features of various data types, tested different combinations of features: I) personal information only, II) admission note only, III) vital signs only, I&III) personal information and vital signs, etc.	AUC of 0.88.	Random forest model, logistic regression CV, Bernoulli NB, gradient boosting classifier, NLP (details not mentioned)	Yes	Yes
22. Arroyo-Gallego, T.,Ledesma-Carbayo, M. J.,Butterworth, I.,Matarazzo, M.,Montero-Escribano, P.,Puertas-Martin, V.,Gray, M. L.,Giancardo, L.,Sanchez-Ferro, A.	Detecting Motor Impairment in Early Parkinson's Disease via Natural Typing Interaction With Keyboards: Validation of the neuroQWERTY Approach in an Uncontrolled At-Home Setting.	2018	To extend the in-clinic implementation to an at-home implementation by validating the applicability of the neuroQWERTY approach in an uncontrolled at-home setting, using the typing data from subjects' natural interaction with their laptop to enable remote and unobtrusive assessment of PD signs.	Timing information of any keyboard input.	Details about the AI system not reported.	AUC of 0.76 - 0.83	neuroQWERTY	Yes	Yes

23. Pesko, M. F.,Gerber, L. M.,Peng, T. R.,Press, M. J.	Home Health Care: Nurse-Physician Communication, Patient Severity, and Hospital Readmission	2018	To evaluate whether communication failures between home health care nurses and physicians during home care (after hospital discharge) are associated with hospital readmission.	Patient characteristics including comorbidities (chronic conditions from the year prior), age, gender, race, and dual eligibility for Medicare and Medicaid. Moreover, patient clinical and functional health, cognitive function, English language, county of residence, and caregiver support i.e., information on nurse characteristics, including the gender, payment structure, years of experience, and highest degree completed, and the specialty type of the physician responsible for coordinating the episode of home health care.	Linear regression models and a propensity score matching approach were used to assess the relationship between communication failure and readmission. Natural language processing (NLP) was applied to free-text data in electronic medical records to identify failures in communication between home health nurses and physicians. The details on NLP were not provided.	32.6 % of the mean	Linear Regression, natural language processing	No	No
24. Zayas, C. E.,He, Z.,Yuan, J.,Maldonado-Molina, M.,Hogan, W.,Modave, F.,Guo, Y.,Bian, J.	Examining Healthcare Utilization Patterns of Elderly Middle-Aged Adults in the United States	2016	To identify high-cost users of healthcare systems and, ineffective utilization patterns to highlight where targeted interventions could be placed to improve care delivery.	Utilization profile of individual with details of care services used.	A novel multi-level framework applying machine learning methods (i.e., random forest regression and hierarchical clustering) to group patients with similar utilization profiles into clusters. Used a vector space model to characterize a patient's utilization profile as the number of visits to different care providers and prescribed medications.	Not reported.	Random forest model with Hierarchical Agglomerative Clustering	No	No
25. Lin, Y.,Huang, S.,Simon, G. E.,Liu, S.	Data-based Decision Rules to Personalize Depression Follow-up	2018	To establish a rule-based method to identify a set of risk predictive patterns from person-level longitudinal disease measurements by integrating the data transformation, rule discovery and rule evaluation.	Electronic health record (EHR) dataset of depression treatment population containing person-level longitudinal Patient Health Questionnaire (PHQ)-9 scores for assessing depression severity.	Establish a general rule-based analytic framework to identify the longitudinal patterns for predicting disease severity in a heterogeneous patient population, and provide actionable knowledge to support the design of adaptive monitoring strategy and apply this framework on a depression treatment population.	Not reported	Logistic regression, support vector model, Rule Fit	Yes	Yes
26. Hertroijs, D. F. L.,Elissen, A. M. J.,Brouwers, Mcgj,Schaper, N. C.,Kohler, S.,Popa, M. C.,Asteriadis, S.,Hendriks, S. H.,Bilo, H. J.,Ruwaard, D.	A risk score including body mass index, glycated haemoglobin and triglycerides predicts future glycaemic control in people with type 2 diabetes	2018	To identify, predict and validate distinct glycaemic trajectories among patients with newly diagnosed type 2 diabetes treated in primary care.	Patients information including HbA1c, systolic blood pressure, diastolic blood pressure, lipid profile LDL cholesterol, HDL cholesterol, total cholesterol and triglycerides), body mass index, urinary albumin-to-creatinine ratio, presence of heart failure, smoking, alcohol consumption, and history of cardiovascular disease in family members aged <60 years (yes/no) was included in the analysis if obtained at any point before diagnosis or a maximum 12 months after diagnosis.	The latent growth mixture modelling identifies systematically the latent trajectories of glycemic control (5-year trajectory). Three different glycemic trajectories were identified: (1) stable, adequate glycemic control (2) improved glycemic control and (3) deteriorated glycemic control.	AUC of 0.96	Latent growth mixture modelling	No	Yes

27. Kop, R.,Hoogendoorn, M.,Teije, A. T.,Buchner, F. L.,Slottje, P.,Moons, L. M.,Numans, M. E.	Predictive modeling of colorectal cancer using a dedicated pre-processing pipeline on routine electronic medical records	2016	To propose a medical pre-processing pipeline to handle problems such as their temporal, inaccurate and incomplete nature of EMR data. The pipeline was used to generate predictive models for prediction of occurrence of colorectal cancer.	Dataset merged six anonymized (GP recording systems) datasets originating from three urban regions in the Netherlands. Data of patient's age, gender, details of GP consultations (411 million records in total), drug prescriptions (423 million), specialist or additional diagnostic procedure referrals (44.4 million), and lab test outcomes (422 million), over a period of five years.	Presented new pre-processing pipeline and integrated it with other machine learning methods (i.e., classification and regression tree (CART), logistic regression, and random forest).	AUC 0.891	Classification & regression tree, logistic regression, random forest model	No	Yes
28. Denny, J. C.,Choma, N. N.,Peterson, J. F.,Miller, R. A.,Bastarache, L.,Li, M.,Peterson, N. B.	Natural language processing improves identification of colorectal cancer testing in the electronic medical record	2012	To use Electronic Health Record data to identify patients with prior colorectal cancer testing.	Colorectal cancer tests records	A clinical natural language processing system was modified to identify 4 colorectal cancer tests (colonoscopy, flexible sigmoidoscopy, fecal occult blood testing, and double contrast barium enema) within electronic clinical documentation. Text phrases in clinical notes referencing colorectal cancer tests were interpreted by the system to determine whether testing was planned or completed and to estimate the date of completed test.	Recall 93-95%, Precision 88- 94%	KMCI system	Yes	No
29. Zhou, S. M.,Fernandez-Gutierrez, F.,Kennedy, J.,Cooksey, R.,Atkinson, M.,Denaxas, S.,Siebert, S.,Dixon, W. G.,O'Neill, T. W.,Choy, E.,Sudlow, C.,U. K. Biobank Follow-up,Outcomes, Group,Brophy, S.	Defining Disease Phenotypes in Primary Care Electronic Health Records by a Machine Learning Approach: A Case Study in Identifying Rheumatoid Arthritis	2016	1) To use data-driven method to examine clinical codes (risk factors) of a medical condition in primary care electronic health records (EHRs) that can accurately predict a diagnosis of the condition in secondary care EHRs. 2) To develop and validate a disease phenotyping algorithm for rheumatoid arthritis using primary care EHRs.	Primary care EHRs for RA	A machine learning based scheme was used to identify patients with rheumatoid arthritis from primary care EHRs via the following steps: i) selection of variables by comparing relative frequencies of Read codes in the primary care dataset associated with disease case compared to non-disease control (disease/non-disease based on the secondary care diagnosis); ii) reduction of predictors/associated variables using a Random Forest method, iii) induction of decision rules from decision tree model.	Overall accuracy of 92.29%	Random forest model, decision tree model	Yes	Yes

30. Hoogendoorn, M.,Szolovits, P.,Moons, L. M. G.,Numans, M. E.	Utilizing uncoded consultation notes from electronic medical records for predictive modeling of colorectal cancer	2016	To extract valuable predictors from uncoded consultation notes and study whether they can help to improve predictive performance that was evaluated in the context of predictive modeling for colorectal cancer.	Patients information regarding code of symptoms and/or diagnoses during the consultation/patient visit, assigned according to the coding convention used in the dataset (ICPC) coding standard, medication prescribed, including the dosage, coding scheme applied for medication is the Anatomical Therapeutic Chemical (ATC) scheme, any form of lab measurement, the coding scheme is specific for the general practitioner information system from which the dataset was exported, referrals to secondary care, again coded in an information system specific way, and consultation notes.	Developed a new natural language processing (NLP) pipeline. The natural language processing model was used to extract predictors from the consultation notes i.e. a bag of words-based approach and topic modelling (i.e. LDA and HDP--both uses Baysian leaning). The model can match the un-coded consultation notes with medical ontology in the context of predictive modelling for colorectal cancer.	AUC of 0.870 versus 0.831 & AUC of 0.896 versus 0.882	LDA, HDP, Natural language processing	No	Yes
31. Huang, D.,Wu, Z.	Forecasting outpatient visits using empirical mode decomposition coupled with back-propagation artificial neural networks optimized by particle swarm optimization	2017	To develop a hybrid model to forecast outpatient visits on the basis of monthly numbers.	Number of outpatient visits is released from the hospital information section.	Developed a hybrid model for predicting the trend of outpatient visit. The artificial neural networks were optimized by particle swarm optimization (to optimize the weights and thresholds of back-propagation artificial neural networks) to forecast outpatient visits. To compare the results with other methods a simulation is performed.	Not reported	Three-layer back-propagation artificial neural network optimised by particle swarm optimization.	No	No
32. McFadden, P.,Crim, A.	Comparison of the Effectiveness of Interactive Didactic Lecture Versus Online Simulation-Based CME Programs Directed at Improving the Diagnostic Capabilities of Primary Care Practitioners	2016	To compare the effectiveness of two approaches to CME training directed at improving the primary care practitioner’s diagnostic capabilities against seven common and important causes of joint pain.	Practicing primary care providers' data	Details about the AI system not mentioned	$P < .02$; Cohen d, effect size = 0.79	AI-driven diagnostic training simulator/tutor called knowledge-based inference tool	No	No
33. Karystianis, G.,Sheppard, T.,Dixon, W. G.,Nenadic, G.	Modelling and extraction of variability in free-text medication prescriptions from an anonymised primary care electronic medical record research database	2016	To develop a novel model and automated text-mining method to extract detailed structured medication information from free-text prescriptions and explore their variability (e.g., optional dosages) in primary care research databases.	Free-text prescription rubrics that general practitioners have used to instruct administration of medications.	Developed a text mining system that relies on rules to extract such structured information from prescription free-text dosage instructions. The system was applied to medication prescriptions from an anonymized primary care electronic record database.	Overall accuracy of 91 %	Rule-based system	No	No

34. Xu, G.,Player, P.,Shepherd, D.,Brunskill, N. J.	Identifying acute kidney injury in the community--a novel informatics approach	2016	To test the utility of novel informatics software to identify patients with acute kidney injury in the community.	Medical data of high risk and low risk patients in one urban practice	IMPAKT-EVOLVE-AKI is a web based software tool that can be downloaded to be run in-practice. The IMPAKT- EVOLVE-AKI software uses Morbidity Information Query and Export Syntax (MIQUEST) search methodology to execute queries and extract data from general practice computer systems.	Not reported	Expert system (IMPAKT-EVOLVE -AKI software)	No	Yes
35. MacRae, J.,Love, T.,Baker, M. G.,Dowell, A.,Carnachan, M.,Stubbe, M.,McBain, L.	Identifying influenza-like illness presentation from unstructured general practice clinical narrative using a text classifier rule-based expert system versus a clinical expert	2015	To describe the development and testing of a rule based expert-system to identify the presentation of ILI within general practice from routinely recorded clinical narrative.	Clinical data (e.g., sore throat, temperature, runny nose).	Rules were assessed using pattern matching heuristics on routine clinical narrative.	98.2 % specificity and 90.2 % sensitivity	Pattern matching heuristics	No	Yes
36. Patel, R.,Jayatilleke, N.,Broadbent, M.,Chang, C. K.,Foskett, N.,Gorrell, G.,Hayes, R. D.,Jackson, R.,Johnston, C.,Shetty, H.,Roberts, A.,McGuire, P.,Stewart, R.	Negative symptoms in schizophrenia: a study in a large clinical sample of patients using a novel automated method	2015	To identify negative symptoms in the clinical records of a large sample of patients with schizophrenia using natural language processing and assess their relationship with clinical outcomes.	Electronic health record of patients with schizophrenia	Used NLP to detect statements in the correspondence fields of clinical records to determine references to prespecified negative symptoms.	Precision statistics above 0.80	SVM with Natural language processing	No	No
37. MacRae, J.,Darlow, B.,McBain, L.,Jones, O.,Stubbe, M.,Turner, N.,Dowell, A.	Accessing primary care Big Data: the development of a software algorithm to explore the rich content of consultation records	2015	To develop a natural language processing software inference algorithm to classify the content of primary care consultations using electronic health record Big Data and subsequently test the algorithm's ability to estimate the prevalence and burden of childhood respiratory illness in primary care.	Consultation records	A natural language processing software inference algorithm that analyses the content of clinical consultation records, diagnostic classifications and prescription information, is able to classify child-GP consultations related to respiratory conditions with similar accuracy to clinical experts.	Sensitivity of 0.72 (95% CI 0.67 to 0.78) and a specificity of 0.95 (95% CI 0.93 to 0.98)	The Child Respiratory Algorithm	No	No
38. Gu, Y.,Kennelly, J.,Warren, J.,Nathani, P.,Boyce, T.	Automatic Detection of Skin and Subcutaneous Tissue Infections from Primary Care Electronic Medical Records	2015	To explore the feasibility and performance of automatic detection of skin and subcutaneous tissue infections occurrences and recurrences by analysing ambient primary care electronic medical records (EMR).	EMR data (demographic information, laboratory testing results, diagnoses, notes, and prescriptions).	A skin and subcutaneous tissue infections identification algorithm was developed examining EMR data for skin swab tests, diagnoses (READ codes) and textual clinical notes.	The F1 score of the analysis algorithm is 0.76	Natural language processing (details not mentioned)	No	Yes

39. Press, M. J.,Gerber, L. M.,Peng, T. R.,Pesko, M. F.,Feldman, P. H.,Ouchida, K.,Sridharan, S.,Bao, Y.,Barron, Y.,Casalino, L. P.	Post discharge Communication Between Home Health Nurses and Physicians: Measurement, Quality, and Outcomes	2015	To use natural language processing of text from electronic medical records to identify failed communication attempts between home health nurses and physicians, to identify predictors of communication failure, and to assess the association between communication failure and hospital readmission.	Data from Medicare beneficiaries with congestive heart failure.	NLP algorithm to determine the success of each communication attempt by interpreting the physician name and free-text comments that the nurse entered in the EMR.	kappa = 0.850, P < .001	Natural language processing (details not mentioned)	No	No
40. Murtaugh, M. A.,Gibson, B. S.,Redd, D.,Zeng-Treitler, Q.	Regular expression-based learning to extract bodyweight values from clinical notes	2015	To develop and validate a learning algorithm that would extract bodyweight related measures from clinical notes in the Veterans Administration (VA) Electronic Health Record to complement the structured data used in clinical research.	Outpatient primary care notes that were annotated by two annotators.	Developed the Regular Expression Discovery Extractor (REDEx), a supervised learning algorithm that generates regular expressions from a training set. The regular expressions generated by REDEx were then used to extract the numerical values of interest.	Accuracy = 98.3%, Precision = 98.8%, Recall = 98.3%, F = 98.5%	REDEx algorithm	No	No
41. Anderson, H. D.,Pace, W. D.,Brandt, E.,Nielsen, R. D.,Allen, R. R.,Libby, A. M.,West, D. R.,Valuck, R. J.	Monitoring suicidal patients in primary care using electronic health records	2015	To monitor suicide ideation or attempts that are documented in EHRs.	Patient age and sex, ICD-9 coded diagnoses from the problems list, a clinician-populated notes field, and medications ordered.	Used retrospective analyses of de-identified EHR data from a distributed health network of primary care organizations to estimate the frequency of using diagnostic codes to record suicidal ideation and attempts. A clinician notes field processed using natural language processing; a suicidal ideation item on a patient-reported depression severity instrument (9-item Patient Health Questionnaire); and diagnostic codes from the EHR.	Not reported	Rule-based system	No	No
42. Kerr, G. S.,Richards, J. S.,Nunziato, C. A.,Patterson, O. V.,DuVall, S. L.,Aujero, M.,Maron, D.,Amdur, R.	Measuring physician adherence with gout quality indicators: a role for natural language processing	2015	To evaluate physician adherence with gout quality indicators for medication use and monitoring, and behavioral modification (BM).	Data such as gout-specific dietary restrictions, weight loss, and alcohol consumption, socio-demographics, comorbidities, and number of rheumatology and primary care visits.	Natural language processing (NLP) was used to analyze clinical narrative data from electronic medical records (EMRs) of overweight gout patients for BM counseling.	Not reported	Rule-based system	No	No
43. Vijayakrishnan, R.,Steinhubl, S. R.,Ng, K.,Sun, J.,Byrd, R. J.,Daar, Z.,Williams, B. A.,deFilippi, C.,Ebadollahi, S.,Stewart, W. F.	Prevalence of heart failure signs and symptoms in a large primary care population identified through the use of text and data mining of the electronic health record	2014	To identify the documentation of the signs and symptoms of HF in the years preceding its diagnosis.	Heart Failure signs and symptoms	Used unstructured information management architecture (UIMA) to identify clinically relevant entities in clinical notes mentioned as a part of the EHR. The entities are subsequently used for information retrieval and data mining of vast information available in the health records.	Not reported	Unstructured information management architecture (UIMA)	No	No
44. Lappenschaar, M.,Hommersom, A.,Lucas, P. J.,Lagro, J.,Visscher, S.,Korevaar, J. C.,Schellevis, F. G.	Multilevel temporal Bayesian networks can model longitudinal change in multimorbidity	2013	To study the course of multimorbidity in the expectation that this would yield new clinical insight.	Clinical data	In this study, the new method of MTBNs was used to precisely capture the qualitative and quantitative time course of chronic cardiovascular multimorbidity in general practices. Bayesian network methods have not been used before in multimorbidity analysis.	Not reported	Bayesian network	No	Yes

45. Klann, J. G.,Anand, V.,Downs, S. M.	Patient-tailored prioritization for a pediatric care decision support system through machine learning	2013	To demonstrate the ability of a Bayesian structure learning method to 'phenotype the population' seen in our primary care pediatric clinics.	Observational data collected by CHICA	Used Bayesian structure learning to build networks of association among previously collected data from our decision support system.	AUC was 0.65	Bayesian network	No	No
46. Afzal, Z.,Engelkes, M.,Verhamme, K. M.,Janssens, H. M.,Sturkenboom, M. C.,Kors, J. A.,Schuemie, M. J.	Automatic generation of case-detection algorithms to identify children with asthma from large electronic health record databases	2013	To use a machine-learning approach to generate and evaluate an automated case-detection algorithm that uses both free-text and coded information to identify asthma cases.	EMR database for asthma patients	The rule-learning program RIPPER was used to generate algorithms to distinguish cases from non-cases. An over-sampling method was used to balance the performance of the automated algorithm to meet our study requirements.	PPV -0.57-0.82	Rule-learning program (RIPPER)	No	Yes
47. Penny, K. I.,Smith, G. D.	The use of data-mining to identify indicators of health-related quality of life in patients with irritable bowel syndrome	2012	To use data-mining models to determine which factors are associated with impaired health-related quality of life using logistic regression, a classification tree and artificial neural networks.	Frequency, duration and severity of symptoms in a cohort of community-based patients with Irritable Bowel Syndrome symptoms.	Logistic regression, a classification tree (CT) and three different artificial neural networks (ANNs) are considered. Logistic regression models are built including only factors which are statistically significant resulting in parsimonious models being developed. A CT within the classification and regression tree model framework is also considered where recursive partitioning is used to split the training records into segments with similar output values. An ANN with 3 hidden layers is included.	Not reported	Logistic regression, ANN, and CT	Yes	No
48. Zhou, L.,Plasek, J. M.,Mahoney, L. M.,Karipineni, N.,Chang, F.,Yan, X.,Chang, F.,Dimaggio, D.,Goldman, D. S.,Rocha, R. A.	Using Medical Text Extraction, Reasoning and Mapping System (MTERMS) to process medication information in outpatient clinical notes	2011	To develop a general natural language processing (NLP) system, called Medical Text Extraction, Reasoning and Mapping System (MTERMS), which encodes clinical text using different terminologies and simultaneously establishes dynamic mappings between them.	Clinical notes	MTERMS applies a modular, pipeline approach flowing from a preprocessor, semantic tagger, terminology mapper, context analyzer, and parser to structure inputted clinical notes.	F-measure of 90.6 and 94.0	MTERMS	Yes	No
49. Ladstatter, F.,Garrosa, E.,Badea, C.,Moreno, B.	Application of artificial neural networks to a study of nursing burnout	2010	To assess whether artificial neural network (ANN) paradigms offer greater predictive accuracy than statistical methodologies, which are commonly used in the field of burnout.	Survey on experience as a nurse in a hospital and interacting with patients	A radial basis function (RBF) network and hierarchical stepwise regression was used to assess burnout.	Compared to the hierarchical stepwise regression analysis, the coefficients of determination were about 37% higher for the RBF network.	A radial basis function (RBF) network and hierarchical stepwise regression = ANN	Yes	No
50. Rennie, T. W.,Roberts, W.	Data mining of tuberculosis patient data using multiple correspondence analysis	2009	To demonstrate the epidemiological use of multiple correspondence analysis (MCA), as applied to tuberculosis (TB) data	TB data from North East London	MCA analyses were performed on the whole dataset, by PCT, and by year of notification.	Not reported	Multiple correspondence analysis	No	No

51. Pakhomov, S. V.,Jacobsen, S. J.,Chute, C. G.,Roger, V. L.	Agreement between patient-reported symptoms and their documentation in the medical record	2008	To determine the agreement between patient-reported symptoms of chest pain, dyspnea and cough and the documentation of these symptoms by physicians in the electronic medical record (EMR).	Symptoms reported by patients on patient provided information forms	Symptoms compared to those identified with natural language processing (NLP) of the text of clinical notes from care providers. Terms that represent the three symptoms were used to search clinical notes electronically with subsequent manual identification of the context (e.g., affirmative, negated, family history) in which they occur.	Kappa statistics were 0.50 (95%CI 0.41-0.59) for chest pain, 0.46 (95%CI 0.37-0.54) for dyspnea and 0.38 (95%CI 0.28-0.48)	Natural language processing (details not reported)	No	No
52. Maizels, M.,Wolfe, W. J.	An expert system for headache diagnosis: the Computerized Headache Assessment tool (CHAT)	2008	To develop and determine the accuracy and utility of a computerized headache assessment tool (CHAT). CHAT was designed to identify all the major primary headache disorders, distinguish daily from episodic types, and recognize medication overuse.	Headache frequency and duration	Details about the AI system not mentioned	Not reported	Expert system (computerized headache assessment tool)	No	Yes
53. Zhu, M.,Chen, W.,Hirdes, J. P.,Stolee, P.	The K-nearest neighbor algorithm predicted rehabilitation potential better than current Clinical Assessment Protocol	2007	To explore the potential to use an automatic, data-driven, machine-learning algorithm in clinical decision making.	Mobility in bed, Transfer, Locomotion in home, Locomotion outside of home, Dressing upper body, Dressing lower body, Eating, toilet use, personal hygiene, bathing, ability to understand others, etc	Used the same covariates in KNN as the ones used by ADLCAP. A random sample of 2,500 clients from the other seven data sets and use it as the training set. This strategy automatically allows KNN to avoid using one's own data to predict itself (and thereby creating a bias toward better prediction).	Lower false positive rate in all but one of the eight regions in the sample, and lower false negative rates in all regions.	KNN	Yes	Yes
54. Tandon, R.,Adak, S.,Kaye, J. A.	Neural networks for longitudinal studies in Alzheimer's disease	2006	To propose a new method of predicting the course of a disease using longitudinal data collected through multiple clinic visits.	Longitudinal data	A back-propagation algorithm, modified for longitudinal data is used to obtain the weight parameters of the MENN. The modified back-propagation algorithm is further embedded in an iterative procedure that estimates the noise variance and the parameters that capture the longitudinal (temporal) correlation structure.	Misclassification rate = 0.13 and relative MSE = 0.35	Mixed effect neural network	Yes	Yes
55. Hoving, C.,Mudde, A. N.,de Vries, H.	Intention to adopt a smoking cessation expert system within a self-selected sample of Dutch general practitioners	2006	To investigate intention to adopt a new smoking cessation expert system as well as outline perceived barriers by general practitioners (GPs)	Data obtained from questionnaires filled by GPs	Details about the AI system not reported	Not reported	Expert system	No	No

56. Goldstein, M. K.,Coleman, R. W.,Tu, S. W.,Shankar, R. D.,O'Connor, M. J.,Musen, M. A.,Martins, S. B.,Lavori, P. W.,Shlipak, M. G.,Oddone, E.,Advani, A. A.,Gholami, P.,Hoffman, B. B.	Translating research into practice: organizational issues in implementing automated decision support for hypertension in three medical centers	2004	To describe the application of a ‘‘sociotechnical’’ approach to integration of ATHENA DSS, a decision support system for the treatment of hypertension, into geographically dispersed primary care clinics.	EMR system patient data	Applied an iterative technical design in response to organizational input and obtained ongoing endorsements of the project by the organization’s administrative and clinical leadership.	Not reported	Expert system (ATHENA DSS)	No	No
57. Taylor, R. J.,Taylor, A. D.,Smyth, J. V.	Using an artificial neural network to predict healing times and risk factors for venous leg ulcers	2002	To identify the risk factors that influence the healing process of venous leg ulcers treated with compression bandaging, in order to predict healing time.	Data of patients with venous ulcers	Artificial neural network (ANN) is an information processing model inspired by the way in which biological nervous systems such as the brain process information. It is composed of a large number of highly interconnected processing components (neurons) which work in unison to solve a particular problem. Use of an ANN technique accurately predicted the healing times.	Not reported	Artificial neural network	No	No
58. Bindels, R.,Winkens, R. A.,Pop, P.,van Wersch, J. W.,Talmon, J.,Hasman, A.	Validation of a knowledge based reminder system for diagnostic test ordering in general practice	2001	To describe the validation of a real-time automated reminder system that assists General Practitioners (GP) in appropriate test ordering.	Medical information of patient	Details about the AI system not reported.	7% results given incorrectly	Expert system (GRIF)	No	No
59. Knab, J. H.,Wallace, M. S.,Wagner, R. L.,Tsoukatos, J.,Weinger, M. B.	The use of a computer-based decision support system facilitates primary care physicians' management of chronic pain	2001	To test whether computer-based decision support (CBDS) could enhance the ability of primary care physicians (PCPs) to manage chronic pain.	Data from chronic pain patients referred by PCPs to a pain clinic.	Details about the AI system not reported.	Mean combined appropriateness score of 5.5 ±0.1	Expert system (Chronic pain algorithm)	No	No
60. Smith, H. R.,Ashton, R. E.,Brooks, G. J.	Initial use of a computer system for assisting dermatological diagnosis in general practice	2000	To describe skin lesions accurately using DERMIS.	Dermatological data of patients	Details about the AI system not reported.	Not reported.	Expert system (DERMIS)	No	Yes
61. Hung, J.,Posey, J.,Freedman, R.,Thorton, T.	Electronic surveillance of disease states: a preliminary study in electronic detection of respiratory diseases in a primary care setting	1998	To describe the creation of a computerized surveillance system using historical information derived from automated expert system acquisition.	Historical medical information.	Details about the AI system not reported.	Not reported	Expert system	No	Yes

62. Abdel-Aal, R. E.,Mangoud, A. M.	Modeling obesity using abductive networks	1997	To use abductory inductive mechanism (AIM) abductive machine learning to analyse medical health surveys (as an alternative to conventional regression analysis and neural modeling) to model obesity.	Age, weight, and height, the model uses both the systolic and diastolic blood pressures and the fasting blood sugar. The body mass index diagnosed heart disease, and cholesterol level also feature as inputs.	The used version of AIM supports the following main elements: (i) A white element which consists of a constant plus the linear weighted sum of all outputs of the previous layer; (ii) Single, double, and triple elements which implement a third-degree polynomial expression with all possible cross-terms for one, two, and three inputs respectively.	Error of 7.5% at the 90% confidence limits, accuracy as high as 99%	AIM abductive network	No	Yes
63. Ridderikhoff, J.,van Herk, E.	A diagnostic support system in general practice: is it feasible?	1997	To study the feasibility of a diagnostic support system in general practice	Physicians' diagnosis data	An expert system called Diagnostic decision support system was used.	Diagnostic accuracy was 43%.	Expert system (Diagnostic decision support system)	No	Yes
64. Gautier, V.,Redier, H.,Pujol, J. L.,Bousquet, J.,Proudhon, H.,Michel, C.,Daures, J. P.,Michel, F. B.,Godard, P.	Comparison of an expert system with other clinical scores for the evaluation of severity of asthma	1996	To compare a new score called Artificial Intelligence score (AI score), produced by an expert system (Asthmaexpert) with three other scores.	Patient's medical data pertaining to asthma.	Details about the AI system not reported.	Kappa=18, 28 and 10% for Aas, Hargreave and Brooks, respectively	Expert system (Asthmaexpert)	Yes	Yes
65. Balas, E. A.,Li, Z. R.,Spencer, D. C.,Jaffrey, F.,Brent, E.,Mitchell, J. A.	An expert system for performance-based direct delivery of published clinical evidence	1996	To develop a system for clinical performance improvement through rule-based analysis of medical practice patterns and individualized distribution of published scientific evidence.	Clinical data	Details about the AI system not reported.	Not reported	Expert system (Quality Feedback Expert System)	No	No
66. Haslam, N.,Beck, A. T.	Categorization of major depression in an outpatient sample	1993	To categorise major depression in patients	Symptoms like depressed mood, pessimism, sense of failure, guilt feelings, self-accusation, etc.,	Details about the AI system not reported.	Not reported	COBWEB/3 model	No	Yes
67. Jordan, P.,Shedden-Mora, M. C.,Löwe, B.	Predicting suicidal ideation in primary care: An approach to identify easily assessable key variables	2018	1) To obtain predictors of suicidal ideation, which can also be used for an indirect assessment of suicidal ideation . 2) To create a classifier for SI based on variables of the Patient Health Questionnaire and sociodemographic variables, 3) to obtain an upper bound on the best possible performance of a predictor based on those variables.	Cross-sectional data from a broad screening assessment. Having severe somatic or psychiatric disease, severe cognitive disabilities, being younger than 18 years old, having impaired vision, and insufficient German language skills defined exclusion criteria.	Advanced methods of machine learning (Classifier trees, Support vector machine(SVM) , Neural Networks) were used to derive the prediction equation. Various classifiers were applied and the area under the curve (AUC) was computed as a performance measure.	AUCs around 0.87 for classifiers	Classification trees, SVM, Neural Networks, Fisher's linear discriminant function	Yes	Yes
68. Monahan, M.,Jowett, S.,Lovibond, K.,Gill, P.,Godwin, M.,Greenfield, S.,Hanley, J.,Hobbs, F. D. R.,Martin, U.,Mant, J.,McKinstry, B.,Williams, B.,Sheppard, J. P.,McManus, R. J.	Predicting out-of-office blood pressure in the clinic for the diagnosis of hypertension in primary care: An economic evaluation	2018	To predict masked and white coat hypertension based on patient characteristics and clinic blood pressure.	Clinical data such as Prevalence of true hypertension in population suspected of having hypertension, Prevalence of masked hypertension in screening population, Diagnosis inputs	A Markov cost–utility cohort model was developed to compare diagnostic strategies: the PROOF-BP approach, including those who receive ambulatory BP monitoring as guided by the algorithm, compared with current standard diagnostic strategies combined with further monitoring.	Not reported	Markov cost-utility model	Yes	Yes

69. Thakur, S.,Dharavath, R.	Artificial neural network based prediction of malaria abundances using big data: A knowledge capturing approach	2018	To determine the malaria abundance using clinical and environmental variables with Big Data from the geographical location of Khammam district, Telangana, India.	Total no. of cases, Average age, %male, %female, Average Temperature (°C), Average Rainfall(mm)	To investigate the competence of artificial neural network process, weight is attached to determine the corresponding neuron and data pass signals between neurons. These signals are processed as an integrated function which combines the signals and proceeds for activation function and finally passes the output.	Ranges from 18% to 117%. - RMPSE Error	Artificial neural network	No	Yes
70. Selskyy, P.,Vakulenko, D.,Televiak, A.,Veresiuk, T.	On an algorithm for decision-making for the optimization of disease prediction at the primary health care level using neural network clustering	2018	To develop an algorithm to optimize the decision-making prognosis of disease at the primary health care level based on information methods.	The data used for analysis originated from the survey results of 63 patients with hypertension in educational and practical centers of primary health care (EPCPHC) of Ternopil region (Ukraine).	For a deeper analysis and clustering, the neural network approach was used with the NeuroXL Classifier add-in application for Microsoft Excel.	Not reported	Optimizatin using Neural network (NeuroXL classifier)	No	Yes
71. Levy, B.,Hogan, J.,Hess, C.,Greenspan, S.,Hogan, M.,Gable, S.,Falcon, K.,Elber, A.,O'Connor, M.,Driscoll, D.,Hashmi, A.	Machine Learning Detection of Cognitive Impairment in Primary Care	2018	To evaluate the validity of a screening procedure that had been specifically designed to impose minimal burden on the clinic.	Data from computerized cognitive test (the CNS Screen)	Analyses employed a machine learning model of Support Vector Machines to classify non-symptomatic subjects from a primary care clinic and hospitalized psychiatric patients with mild cognitive impairment, based on the screening data.	Probability of 0.945	SVM	No	Yes
72. Janssen, K. J. M.,Siccama, I.,Vergouwe, Y.,Koffijberg, H.,Debray, T. P. A.,Keijzer, M.,Grobbee, D. E.,Moons, K. G. M.	Development and validation of clinical prediction models: Marginal differences between logistic regression, penalized maximum likelihood estimation, and genetic programming	2012	TO compare the accuracy of a model that predicts the presence of deep venous thrombosis (DVT) when developed by four different methods.	Primary care patients suspected of DVT	The models were developed by logistic regression, logistic regression with shrinkage by boot- strapping techniques, logistic regression with shrinkage by penalized maximum likelihood estimation, and genetic programming. The accuracy of the models was tested by assessing discrimination and calibration.	ROC for logistic regression without shrinkage was 0.904 (95% confidence interval [CI]: 0.885e0.922), for logistic regression with shrinkage was 0.904 (95% CI: 0.885e0.922).for logistic regression with shrinkage by penalized maximum likelihood estimation was 0.902 (95% CI: 0.883e0.921), for GP was 0.910 (95% CI: 0.893e0.928)	Logistic regression, logistic regression with shrinkage by boot- strapping techniques, logistic regression with shrinkage by penalized maximum likelihood estimation, and genetic programming.	Yes	Yes

73. Ting, S. L.,Kwok, S. K.,Tsang, A. H. C.,Lee, W. B.	A hybrid knowledge-based approach to supporting the medical prescription for general practitioners: Real case in a Hong Kong medical center	2011	1) To provide general practitioners with medication advice 2) To suggest a range of medicines for specific medical conditions by taking into consideration the collective pattern as well as the individual preferences of physicians' prescription decisions.	A set of historical medical prescription records stored in a knowledge base	A hybrid approach is described that uses a combination of case-based reasoning (CBR) and Bayesian reasoning. In the CBR process, all the previous knowledge retrieved via similarity measures is made available for the reference of physicians as to what medicines have been prescribed (to a particular patient) in the past. After obtaining the results from CBR, Bayesian reasoning is then applied to model the prescription experience of all physicians within the organization. By comparing the two sets of results, more refined recommendations on a range of medicines are suggested along with the ranking for each recommendation.	Not reported	CBR and Bayesian reasoning	No	No
74. Ting, S. L.,Wang, W. M.,Kwok, S. K.,Tsang, A. H. C.,Lee, W. B.	RACER: Rule-Associated CasE-based Reasoning for supporting General Practitioners in prescription making	2010	To develop a revised Case-based Reasoning (CBR) mechanism, named Rule-Associated CasE-based Reasoning (RACER), which integrates CBR and association rules mining for supporting General Practitioners prescription.	The diagnosis, the medical data recorded in EMR consisting of all the examination data and patient particular information which is voluminous and heterogeneous. Data is preprocessed by selecting attributes or features which are useful for prescription making. (Examination data of disease determined by the GP).	RACER methodology has 3 main parts: cases retrieval, association rules mining, and suggestions combination. First, a new case (the diagnosis) is codified based on a predefined EMR. The codified new case is then processed by comparing with the previous cases retained in the knowledge base. Association rules mining and case retrieval are then applied. Association rules mining is used to extract the most interesting association rules based on support and confidence measure.	Not reported	RACER, which integrates CBR and association rules mining	Yes	No
75. Doukidis, G. I.,Forster, D.	The Potential for Computer-Aided Diagnosis of Tropical Diseases In Developing-Countries - An Expert System Case-Study	1990	To develop and implement ESTROPID, an Expert system on tropical diseases, to assist paramedical staff during training and in clinical practice.	Clinical data	Details about the AI system not reported	Not reported	Expert system (Estropid)	No	Yes
76. Tariq, Amina,Westbrook, Johanna,Byrne, Mary,Robinson, Maureen,Baysari, Melissa T.	Applying a human factors approach to improve usability of a decision support system in tele-nursing	2017	To evaluate usability of a decision support system for telephone triage nurses.	Data gathered using telephone triage	Applied a multi-method human factors approach to evaluate the usability of decision support software used by Healthdirect Australia nurses during telephone triage. Methods included: (1) stakeholder discussions; (2) heurstic analysis by two independent experts across ten usability heuristics; and (3) interviews with system end users.	Not reported	Expert system (Call Enhance Call Centre)	No	No
77. Sayadi, Mehrab,Zibaeenezhad, Mohammadjavad,Taghi Ayatollahi, Seyyed Mohammad	Simple Prediction of Type 2 Diabetes Mellitus via Decision Tree Modeling	2017	To present a simple model for predicting Type 2 Diabetes using decision tree modeling.	Data included routine information, such as age, gender, Body Mass Index, family history of diabetes, and systolic and diastolic blood pressure.	Decision tree technique and J48 algorithm were applied using the WEKA software (version 3.7.5, New Zealand).	AUC were 87% and 89%	Decision tree	Yes	Yes

78. Aberdeen, J.,Bayer, S.,Clark, C.,Keybl, M.,Tresner-Kirsch, D.	An annotation and modeling schema for prescription regimens	2019	To describe the annotation schema, the curation of a corpus of prescription instructions through a manual annotation effort, and initial experiments in modeling and automated generation of TranScriptML representations.	Ambulatory prescriptions	Developed the representation schema as a novel set of semantic tags for prescription concept categories (e.g., frequency); each tag label is defined with an accompanying attribute framework in which the meaning of tagged concepts can be specified in a normalized fashion. and used Conditional Random Field machine learning and various other methods to train automated annotation models based on the manual annotations.	Accuracy above 0.9.	Conditional random field (TranScriptML)	No	No
79. Abramoff, M. D.,Lavin, P. T.,Birch, M.,Shah, N.,Folk, J. C.	Pivotal trial of an autonomous AI-based diagnostic system for detection of diabetic retinopathy in primary care offices	2018	To conduct a pivotal trial of an AI system to detect diabetic retinopathy (DR) in people with diabetes.	Data of asymptomatic persons, ages of 22 and older, who had been diagnosed with diabetes and had not been previously diagnosed with DR.	The autonomous AI system, IDx-DR, has two core algorithms, an Image Quality AI-based algorithm, and the Diagnostic Algorithm proper. The complete AI system was locked before the start of this study.	Sensitivity of 87.2% (95% CI, 81.8–91.2%) (>85%), specificity of 90.7% (95% CI, 88.3–92.7%) (>82.5%), and imageability rate of 96.1% (95% CI, 94.6–97.3%)	Image quality algorithm, convolutional neural networks	No	Yes
80. Adams, Ellise	Toward Evidence-Based Practice. Predicting Common Maternal Postpartum Complications: Leveraging Health Administrative Data and Machine Learning	2019	To predict the risk of common maternal postpartum complications requiring an inpatient episode of care.	Maternal data from the beginning of gestation up to and including the delivery, and neonatal data recorded at delivery, were used to predict postpartum complications.	Gradient boosted trees were used with five-fold cross- validation to compare model performance. The best performing models for each outcome were then assessed in the independent validation data	AUC = 0.879, 95% CI 0.846–0.912 & AUC = 0.856, 95% CI 0.838–0.873	Gradient boosted tree model	No	Yes
81. Ben-Sasson, Ayelet,Robins, Diana L.,Yom-Tov, Elad	Risk Assessment for Parents Who Suspect Their Child Has Autism Spectrum Disorder: Machine Learning Approach	2018	To test the feasibility of assessing autism spectrum disorder risk in parental concerns from Web-based sources, using automated text analysis tools and minimal standard questioning.	Parents reported their concerns online, and completed an autism spectrum disorder-specific screener, the Modified Checklist for Autism in Toddlers-Revised, with Follow-up (M-CHAT-R/F), and a broad developmental screener, the Ages and Stages Questionnaire .	Algorithm predicted autism spectrum disorder risk using a combination of the parent's text and a single screening question, selected by the algorithm to enhance prediction accuracy.	Not reported	Machine learning model (not specified by authors)	Yes	Yes
82. Betancourt-Hernandez, M.,Viera-Lopez, G.,Serrano-Munoz, A.	Automatic Diagnosis of Rheumatoid Arthritis From Hand Radiographs Using Convolutional Neural Networks	2018	To develop a system based on Artificial Intelligence (AI), using Convolutional Neural Networks (CNN) for the automatic detection of Rheumatoid Arthritis from hand radiographs.	Gray-scale digital radiographs of both hands, used by medical specialist in their diagnosis of RA.	For the implementation of neural networks, the open source library Keras was used. OpenCV and Numpy modules were used to handle and process the images.	Accuracy of 100 %	Convolutional neural network	No	Yes

83. Burton, R. J.,Albur, M.,Eberl, M.,Cuff, S. M.	Using artificial intelligence to reduce diagnostic workload without compromising detection of urinary tract infections	2019	To see if machine learning models can be used in predicting whether urine samples are likely to require bacterial culture.	Urine reports	A heuristic model using a combination of white blood cell count and bacterial count, and a machine learning approach testing three algorithms (Random Forest, Neural Network, Extreme Gradient Boosting) whilst factoring in independent variables including demographics, historical urine culture results, and clinical details provided with the specimen.	Sensitivity of 95%	Random Forest, Neural Network, Extreme Gradient Boosting	Yes	No
84. Chen, Y. F.,Lin, C. S.,Hong, C. F.,Lee, D. J.,Sun, C.,Lin, H. H.	Design of a Clinical Decision Support System for Predicting Erectile Dysfunction in Men Using NHIRD Dataset	2019	To predict erectile dysfunction in men using clinical decision support system,	Patient's health data regarding ED and other comorbidities.	Integrated genetic algorithm (GA) and support vector machine was adopted to design the CDSSs with 2 experiments of independent training and testing conducted to verify their effectiveness.	Accuracy, sensitivity, specificity, g-mean, and area under ROC curve (AUROC) of 74.72-76.65%, 72.33-83.76%, 69.54-77.10%, 0.7468-0.7632, and 0.766-0.817	Integrated genetic algorithm and support vector machine	Yes	Yes
85. Hill, N. R.,Ayoubkhani, D.,McEwan, P.,Sugrue, D. M.,Farooqui, U.,Lister, S.,Lumley, M.,Bakhai, A.,Cohen, A. T.,O'Neill, M.,Clifton, D.,Gordon, J.	Predicting atrial fibrillation in primary care using machine learning	2019	To develop an implementable risk model that is contemporaneous and informed by routinely collected patient data, reflecting the real-world pathology of atrial fibrillation.	Medical data of people without a history of atrial fibrillation.	Models evaluated included published risk models (Framingham, ARIC, CHARGE-AF), machine learning models, which evaluated baseline and time-updated information (neural network, LASSO, random forests, support vector machines), and Cox regression.	Time-varying neural networks achieving an AUROC of 0.827	Neural network, LASSO, random forests, support vector machines), and Cox regression.	Yes	Yes
86. Kanagasingam, Y.,Xiao, D.,Vignarajan, J.,Preetham, A.,Tay-Kearney, M. L.,Mehrotra, A.	Evaluation of Artificial Intelligence-Based Grading of Diabetic Retinopathy in Primary Care	2018	To describe the performance of an AI system for diabetic retinopathy deployed in a primary care practice.	Data of patients with diabetes	The tele-retinal and AI system includes a color fundus camera (Canon CR-2 AF), a cloud computing server, and a web application server.	Specificity was 92% (95% CI, 87%-96%)	Convolutional neural network	No	Yes
87. Perveen, S.,Shahbaz, M.,Keshavjee, K.,Guergachi, A.	Prognostic Modeling and Prevention of Diabetes Using Machine Learning Technique	2019	To explore the potential role of a Hidden Markov Model (HMM), a machine learning technique, in validating the performance of the Framingham Diabetes Risk Scoring Model (FDRSM), a well-respected prognostic model.	Electronic Medical Record (EMR) data.	HMM is a parametric machine learning technique, HMMs represent probability distributions over sequences of observations.	AUROC 86.9%	Hidden Markov model	No	Yes
88. Ursenbach, J.,O'Connell, M. E.,Neiser, J.,Tierney, M. C.,Morgan, D.,Kosteniuk, J.,Spiteri, R. J.	Scoring algorithms for a computer-based cognitive screening tool: An illustrative example of overfitting machine learning approaches and the impact on estimates of classification accuracy	2019	To study the impact of Scoring algorithms for a computer-based cognitive screening tool.	Independent diagnoses of mild cognitive impairment.	CAMCI assesses five cognitive domains using eight tasks: attention assessed with visual distractor and forward digit span tasks, working memory with a backward digit span task, verbal memory assessed by free recall and a recognition task, visual memory with a recognition task, and executive function assessed with a go/no go task.	Decision tree sensitivity (0.94, specificity 0.94), logistic regression model (sensitivity 0.72, specificity 0.80)	Decision tree model, logistic regression model	Yes	Yes

89. Verbraak, F. D.,Abramoff, M. D.,Bausch, G. C. F.,Klaver, C.,Nijpels, G.,Schlingemann, R. O.,van der Heijden, A. A.	Diagnostic Accuracy of a Device for the Automated Detection of Diabetic Retinopathy in a Primary Care Setting	2019	To determine the diagnostic accuracy in a real-world primary care setting of a deep learning–enhanced device for automated detection of diabetic retinopathy (DR).	Retinal images of people with type 2 diabetes visiting a primary care screening program.	Retinal images of people with type 2 diabetes visiting a primary care screening program were graded by a hybrid deep learning–enhanced device (IDx-DR-EU-2.1; IDx, Amsterdam, the Netherlands), and its classification of retinopathy (vision-threatening [vt]DR, more than mild [mtm]DR, and mild or more [mom]DR) was compared with a reference standard.	The sensitivity/specificity, per the ICDR reference standard, for the device to detect vtDR was 100% (95% CI 77.1– 100)/97.8% (95% CI 96.8–98.5) and mtmDR 79.4% (95% CI 66.5–87.9)/ 93.8% (95% CI 92.1–94.9).	Hybrid deep learning enhanced device	Yes	Yes
90. Wong, E. S.,Schuttner, L.,Reddy, A.	Does machine learning improve prediction of VA primary care reliance?	2020	To examine whether machine learning methods can better predict future reliance on Veterans Administration (VA) primary care compared with traditional statistical methods.	The primary data on information on utilization of Veterans Administration (VA) health services, demographic data.	Compared the performance of 6 candidate models—logistic regression, elastic net regression, decision trees, random forest, gradient boosting machine, and neural network—in predicting	Mean AUC Logistic regression - 0.891, elastic net- 0.891 , decision trees- 0.873, random forest-0.889, gradient boosting machine-0.892, and neural network-0.885	Logistic regression, elastic net regression, decision trees, random forest, gradient boosting machine, and neural network	Yes	No
