# Supplementary material for: Application of Artificial Intelligence in Community-Based Primary Health Care: Systematic Scoping Review and Critical Appraisal
Source: J Med Internet Res. 2021 Sep 3;23(9):e29839. doi: 10.2196/29839 (PMC8449300; doi:10.2196/29839)
Supplement: Multimedia Appendix 5 [file jmir_v23i9e29839_app5.pdf]

Multimedia Appendix 5: Risk of bias items for each evaluated study

Authors	Participants	Predictors	Outcome	Analysis
1. (MORENO; LUJAN; ANYO LUJAN; TORRRES RUSINOL *et al.*, 2016; MORENO; LUJÁN; RUSIÑOL; FERNÁNDEZ *et al.*, 2017)	Low	Low	High	Unclear
2. (LIN; BRUNI; FU; MALONEY *et al.*, 2012)	Low	Low	High	High
3. (TRAN; FANG; PHAM; LIN *et al.*, 2018)	Low	Low	Unclear	Unclear
4. (ZHENG; LUO; MERCADO; SY *et al.*, 2018)	Low	Low	High	Low
5. (LAU; WILKINSON; MOORTHY, 2018)	Low	Low	High	Low
6. (JARVIK; GOLD; TAN; FRIEDLY *et al.*, 2018)	Low	Low	High	Low
7. (THABTAH; KAMALOV; RAJAB, 2018)	High	Unclear	High	High
8. (BRAIDO; SANTUS; CORSICO; DI MARCO *et al.*, 2018)	Low	Low	High	High

9. (LUO; SMALL; STEWART; ROY, 2013)	Low	High	Low	Low
10. (MORALES; FLYNN; ZHANG; TRUCCO *et al.*, 2018)	Low	High	High	Low
11. (TOU; YAO; WEI; ZHUANG *et al.*, 2018)	Low	Low	High	Low
12. (ARROYO-GALLEGO; LEDESMA-CARBAYO; BUTTERWORTH; MATARAZZO *et al.*, 2018)	Low	High	Unclear	High
13. (LIN; HUANG; SIMON; LIU, 2018)	Low	Low	High	Unclear
14. (HERTROIJS; ELISSEN; BROUWERS; SCHAPER *et al.*, 2018)	Low	High	Unclear	Low
15. (KOP; HOOGENDOORN; TEIJE; BUCHNER *et al.*, 2016)	Low	Low	Low	Low
16. (ZHOU; FERNANDEZ-GUTIERREZ; KENNEDY; COOKSEY *et al.*, 2016)	Low	High	Unclear	Low

17. (HOOGENDOORN; SZOLOVITS; MOONS; NUMANS, 2016)	Low	High	High	High
18. (XU; PLAYER; SHEPHERD; BRUNSKILL, 2016)	Low	High	Low	Low
19. (MACRAE; LOVE; BAKER; DOWELL *et al.*, 2015)	Low	Low	High	Low
20. (GU; KENNELLY; WARREN; NATHANI *et al.*, 2015)	Low	High	High	High
21. (LAPPENSCHAAR; HOMMERSOM; LUCAS; LAGRO *et al.*, 2013)	Low	High	Low	Low
22. (AFZAL; ENGELKES; VERHAMME; JANSSENS *et al.*, 2013)	Low	Unclear	High	Low
23. (MAIZELS; WOLFE, 2008)	Low	High	Low	High
24. (ZHU; CHEN; HIRDES; STOLEE, 2007)	Low	Unclear	Unclear	Low
25. (TANDON; ADAK; KAYE, 2006)	Low	High	High	High
26. (SMITH; ASHTON; BROOKS, 2000)	Low	High	High	High

27. (HUNG; POSEY; FREEDMAN; THORTON, 1998)	Low	High	Unclear	Low
28. (ABDEL-AAL; MANGOUD, 1997)	Low	Unclear	High	High
29. (RIDDERIKHOFF; VAN HERK, 1997)	High	Unclear	Low	High
30. (GAUTIER; REDIER; PUJOL; BOUSQUET *et al.*, 1996)	Low	High	Unclear	Low
31. (HASLAM; BECK, 1993)	Low	High	Unclear	Low
32. (JORDAN; SHEDDEN-MORA; LÖWE, 2018)	Low	Low	Unclear	Low
33. (MONAHAN; JOWETT; LOVIBOND; GILL *et al.*, 2018)	Low	High	Unclear	Unclear
34. (THAKUR; DHARAVATH, 2018)	Low	Unclear	High	Low
35. (SELSKYY; VAKULENKO; TELEVIAK; VERESIUK, 2018)	Low	Low	Low	High
36. (LEVY; HOGAN; HESS; GREENSPAN *et al.*, 2018)	Low	High	Low	Unclear
37. (JANSSEN; SICCAMA; VERGOUWE;	Low	Unclear	Low	Low

KOFFIJBERG *et al.*, 2012)				
38. (DOUKIDIS; FORSTER, 1990)	Low	High	High	High
39. (SAYADI; ZIBAEENEZHAD; TAGHI AYATOLLAHI, 2017)	Low	Unclear	Low	Low
40. (ABRAMOFF; LAVIN; BIRCH; SHAH *et al.*, 2018)	Low	Unclear	High	Low
41. (ADAMS, 2019)	Low	Unclear	Unclear	Low
42. (BEN-SASSON; ROBINS; YOM-TOV, 2018)	Low	Low	Unclear	Unclear
43. (BETANCOURT-HERNANDEZ; VIERA-LOPEZ; SERRANO-MUNOZ, 2018)	Low	Low	Low	High
44. (CHEN; LIN; HONG; LEE *et al.*, 2019)	Low	Low	Unclear	Low
45. (HILL; AYOUBKHANI; MCEWAN; SUGRUE *et al.*, 2019)	Low	Low	High	Low
46. (KANAGASINGAM; XIAO; VIGNARAJAN; PREETHAM *et al.*, 2018)	Low	Low	High	High

47. (PERVEEN; SHAHBAZ; KESHAVJEE; GUERGACHI, 2019)	Low	Unclear	Low	Unclear
48. (URSENBACH; O'CONNELL; NEISER; TIERNEY *et al.*, 2019)	Low	High	Unclear	Unclear
49. (VERBRAAK; ABRAMOFF; BAUSCH; KLAVER *et al.*, 2019)	Low	High	Low	High
