# Supplementary material for: Application of Artificial Intelligence in Community-Based Primary Health Care: Systematic Scoping Review and Critical Appraisal
Source: J Med Internet Res. 2021 Sep 3;23(9):e29839. doi: 10.2196/29839 (PMC8449300; doi:10.2196/29839)
Supplement: Multimedia Appendix 6 [file jmir_v23i9e29839_app6.pdf]

Multimedia Appendix 6: Risk of Bias table: based on authors' judgements about each risk of bias item

	Participants		Predictors			Outcome						Analysis								
Author	Were appropriate data sources used, e.g: cohort, RCT, or nested case–control study data?	Were all inclusions and exclusions of participants appropriate?	Were predictors defined and assessed in a similar way for all participants?	Were predictor assessments made without knowledge of outcome data?	Are all predictors available at the time the model is intended to be used?	Was the outcome determined appropriately?	Was a prespecified or standard outcome definition used?	Were predictors excluded from the outcome definition?	Was the outcome defined and determined in a similar way for all participants?	Was the outcome determined without knowledge of predictor information?	Was the time interval between predictor assessment and outcome determination appropriate?	Were there a reasonable number of participants with the outcome?	Were continuous and categorical predictors handled appropriately?	Were all enrolled participants included in the analysis?	Were participants with missing data handled appropriately?	Was selection of predictors based on univariable analysis avoided? (For developmental studies only)	Were complexities in the data (e.g., censoring, competing risks, sampling of control participants) accounted for	Were relevant model performance measures evaluated appropriately?	Were model overfitting, underfitting, and optimism in model performance accounted for? (For developmental [illegible]	Do predictors and their assigned weights in the final model correspond to the results from the reported [illegible]
1. (MORENO; LUJAN; ANYO LUJAN; TORRRES RUSINOL *et al.*, 2016; MORENO; LUJÁN; RUSIÑOL; FERNÁNDE	+	+	+	+	+	-	+	+	+	-	-	+	+	+	?	+	+	+	+	+

Z *et al.*, 2017)																				
2. (LIN; BRUNI; FU; MALONEY *et al.*, 2012)	-	+	+	+	+	-	+	+	+	-	+	-	**?**	+	**?**	+	+	+	-	+
3. (TRAN; FANG; PHAM; LIN *et al.*, 2018)	+	+	+	+	+	+	**?**	+	+	+	**?**	+	**?**	+	**?**	+	+	+	-	+
4. (ZHENG; LUO; MERCADO; SY *et al.*, 2018)	+	+	+	+	+	-	+	-	+	-	+	+	+	+	+	+	+	+	+	+
5. (LAU; WILKINSON; MOORTHY, 2018)	+	+	+	+	+	+	+	-	+	-	-	+	+	+	+	+	+	+	+	+
6. (JARVIK; GOLD; TAN; FRIEDLY *et al.*, 2018)	+	+	+	+	+	+	-	+	-	-	+	+	+	+	+	NA	+	+	NA	NA
7. (THABTAH; KAMALOV; RAJAB, 2018)	-	+	**?**	+	**?**	-	+	+	+	-	-	+	-	+	+	+	+	+	+	+
8. (BRAIDO; SANTUS; CORSICO; DI MARCO *et al.*, 2018)	+	+	+	+	+	-	+	-	+	+	+	-	+	+	+	+	+	+	-	+
9. (LUO; SMALL; STEWART; ROY, 2013)	+	+	+	-	-	+	+	+	+	+	+	+	+	+	+	+	+	+	+	+
10. (MORALES; FLYNN; ZHANG; TRUCCO *et al.*, 2018)	+	+	+	-	-	+	+	-	+	-	+	+	+	+	+	NA	+	+	NA	NA
11. (TOU; YAO; WEI; ZHUANG *et al.*, 2018)	+	+	+	+	+	-	+	-	+	+	+	+	+	+	+	NA	+	+	NA	NA
12. (ARROYO-GALLEGO; LEDESMA-CARBAYO;	+	+	-	+	-	+	+	**?**	+	+	**?**	-	+	+	+	NA	+	+	NA	NA

BUTTERWORTH; MATARAZZO *et al.*, 2018)																				
13. (LIN; HUANG; SIMON; LIU, 2018)	+	+	+	+	+	-	+	-	+	-	+	+	+	+	?	+	+	+	?	+
14. (HERTROIJS; ELISSEN; BROUWERS; SCHAPER *et al.*, 2018)	+	+	-	+	+	+	?	?	+	+	+	+	+	+	+	+	+	+	+	+
15. (KOP; HOOGENDOORN; TEIJE; BUCHNER *et al.*, 2016)	+	+	+	+	+	+	+	+	+	+	+	+	+	+	+	+	+	+	+	+
16. (ZHOU; FERNANDEZ-GUTIERREZ; KENNEDY; COOKSEY *et al.*, 2016)	+	+	+	-	-	+	?	+	+	+	?	+	+	+	+	+	+	+	+	+
17. (HOOGENDOORN; SZOLOVITS; MOONS; NUMANS, 2016)	+	+	+	-	+	-	+	-	+	-	+	-	+	+	-	+	+	+	+	+
18. (XU; PLAYER; SHEPHERD; BRUNSKILL, 2016)	+	+	-	+	+	+	+	+	+	+	+	+	+	+	+	NA	+	+	NA	NA
19. (MACRAE; LOVE; BAKER; DOWELL *et al.*, 2015)	+	+	+	+	+	-	+	-	+	+	+	+	+	+	+	+	+	+	+	+
20. (GU; KENNELLY; WARREN; NATHANI *et al.*, 2015)	+	+	+	-	-	+	-	-	+	-	+	+	+	+	+	+	+	-	-	+
21. (LAPPENSCHAAR; HOMMERSOM; LUCAS;	+	+	+	-	-	+	+	+	+	+	+	+	+	+	+	NA	+	+	NA	NA

LAGRO *et al.*, 2013)																				
22. (AFZAL; ENGELKES; VERHAMME; JANSSENS *et al.*, 2013)	+	+	?	+	+	-	+	+	-	+	+	+	+	+	+	NA	+	+	NA	NA
23. (MAIZELS; WOLFE, 2008)	+	+	-	+	+	+	-	+	+	+	-	-	+	+	-	+	+	+	+	+
24. (ZHU; CHEN; HIRDES; STOLEE, 2007)	+	+	?	+	+	+	?	+	?	+	?	+	+	+	+	NA	+	+	NA	NA
25. (TANDON; ADAK; KAYE, 2006)	+	+	+	-	-	-	+	+	+	-	+	-	+	+	+	+	+	+	+	+
26. (SMITH; ASHTON; BROOKS, 2000)	+	+	+	+	-	-	+	-	+	-	+	-	+	+	?	NA	-	+	NA	NA
27. (HUNG; POSEY; FREEDMAN; THORTON, 1998)	+	+	-	+	+	+	?	?	+	+	+	+	+	+	+	+	+	+	+	+
28. (ABDEL-AAL; MANGOUD, 1997)	+	+	+	?	+	+	-	+	+	+	-	-	-	+	-	NA	-	+	NA	NA
29. (RIDDERIKHOFF; VAN HERK, 1997)	-	+	?	+	?	+	+	+	+	+	+	-	+	+	-	+	-	-	?	+
30. (GAUTIER; REDIER; PUJOL; BOUSQUET *et al.*, 1996)	+	+	-	+	+	+	?	+	?	+	+	+	+	+	+	NA	+	+	NA	NA
31. (HASLAM; BECK, 1993)	+	+	+	-	+	?	+	+	+	+	?	+	+	+	+	NA	+	+	NA	NA
32. (JORDAN; SHEDDEN-MORA; LÖWE, 2018)	+	+	+	+	+	+	?	+	+	+	?	+	+	+	+	NA	+	+	NA	NA
33. (MONAHAN; JOWETT; LOVIBOND; GILL *et al.*, 2018)	+	+	+	-	-	+	?	+	+	?	+	+	?	+	?	NA	+	+	NA	NA

34. (THAKUR; DHARAVATH, 2018)	+	+	+	?	+	-	+	+	+	-	-	+	+	+	+	NA	+	+	NA	NA
35. (SELSKYY; VAKULENKO; TELEVIAK; VERESIUK, 2018)	+	+	+	+	+	+	+	+	+	+	+	-	+	+	-	+	+	+	-	+
36. (LEVY; HOGAN; HESS; GREENSPAN *et al.*, 2018)	+	+	-	+	-	+	+	+	+	+	+	-	+	+	?	NA	-	+	NA	NA
37. (JANSSEN; SICCAMA; VERGOUWE; KOFFIJBERG *et al.*, 2012)	+	+	+	?	+	+	+	+	+	+	+	+	+	+	+	+	+	+	+	+
38. (DOUKIDIS; FORSTER, 1990)	+	+	+	-	+	-	+	-	+	+	+	+	-	+	?	+	-	+	-	+
39. (SAYADI; ZIBAEENEZHAD; TAGHI AYATOLLAHI, 2017)	+	+	+	+	?	+	+	+	+	+	+	+	+	+	+	NA	+	+	NA	NA
40. (ABRAMOFF; LAVIN; BIRCH; SHAH *et al.*, 2018)	+	+	+	?	+	+	-	+	-	-	+	+	+	+	+	NA	+	+	NA	NA
41. (ADAMS, 2019)	+	+	?	+	?	?	+	+	+	?	+	+	+	+	+	NA	+	+	NA	NA
42. (BEN-SASSON; ROBINS; YOM-TOV, 2018)	+	+	+	+	+	+	+	+	+	?	+	-	+	+	?	NA	?	+	NA	NA
43. (BETANCOURT-HERNANDEZ; VIERA-LOPEZ; SERRANO-MUNOZ, 2018)	+	+	+	+	+	+	+	+	+	+	+	-	-	+	+	+	+	+	+	+
44. (CHEN; LIN; HONG; LEE *et al.*, 2019)	+	+	+	+	+	+	+	?	+	+	?	+	+	+	+	+	+	+	+	+

45. (HILL; AYOUBKHANI; MCEWAN; SUGRUE *et al.*, 2019)	+	+	+	+	+	-	+	-	+	+	+	+	+	+	+	+	+	+	+	+
46. (KANAGASINGAM; XIAO; VIGNARAJAN; PREETHAM *et al.*, 2018)	+	+	+	+	+	+	-	+	+	+	-	-	+	+	-	NA	-	-	NA	NA
47. (PERVEEN; SHAHBAZ; KESHAVJEE; GUERGACHI, 2019)	+	+	?	+	?	+	+	+	+	+	+	+	+	+	+	NA	?	+	NA	NA
48. (URSENBACH; O'CONNELL; NEISER; TIERNEY *et al.*, 2019)	+	+	-	+	-	+	?	+	+	?	+	+	+	+	+	NA	?	+	NA	NA
49. (VERBRAAK; ABRAMOFF; BAUSCH; KLAVER *et al.*, 2019)	+	+	+	-	+	+	+	+	+	+	+	+	+	+	+	+	+	+	-	+
